# In Vitro Models of Cardiovascular Disease: Embryoid Bodies, Organoids and Everything in Between

**DOI:** 10.3390/biomedicines12122714

**Published:** 2024-11-27

**Authors:** Theodora M. Stougiannou, Konstantinos C. Christodoulou, Dimos Karangelis

**Affiliations:** Department of Cardiothoracic Surgery, Democritus University of Thrace University General Hospital, 68100 Alexandroupolis, Greece; konstantinoschristodoulou@yahoo.gr (K.C.C.); dimoskaragel@yahoo.gr (D.K.)

**Keywords:** cardiovascular, biology, cardiac, organoid, embryoid body, SCME, genetic, disease modeling, pluripotent stem cells, embryonic, development

## Abstract

Cardiovascular disease comprises a group of disorders affecting or originating within tissues and organs of the cardiovascular system; most, if not all, will eventually result in cardiomyocyte dysfunction or death, negatively impacting cardiac function. Effective models of cardiac disease are thus important for understanding crucial aspects of disease progression, while recent advancements in stem cell biology have allowed for the use of stem cell populations to derive such models. These include three-dimensional (3D) models such as stem cell-based models of embryos (SCME) as well as organoids, many of which are frequently derived from embryoid bodies (EB). Not only can they recapitulate 3D form and function, but the developmental programs governing the self-organization of cell populations into more complex tissues as well. Many different organoids and SCME constructs have been generated in recent years to recreate cardiac tissue and the complex developmental programs that give rise to its cellular composition and unique tissue morphology. It is thus the purpose of this narrative literature review to describe and summarize many of the recently derived cardiac organoid models as well as their use for the recapitulation of genetic and acquired disease. Owing to the cellular composition of the models examined, this review will focus on disease and tissue injury associated with embryonic/fetal tissues.

## 1. Introduction

Cardiovascular disease can be defined as a group of disorders affecting the heart and vascular network. A total of 620 million people are living with cardiovascular disease around the world, resulting in an estimated death of 1 in 3 people globally [1]. In general, cardiovascular disease comprises a complex interplay of cells and molecular factors affected by genetic as well as environmental variables; an appropriate model must be thus generally composed of appropriate cell types, recapitulating key aspects of the tissue and disease in question [2,3,4]. Cardiac tissue composition can change depending on developmental stage, often requiring construction of different models at varying maturation stages. Though various types of systems can be produced to recapitulate aspects of cardiac tissue and disease such as animal models, disease modeling carried out in vitro allows for more accurate control of structural and functional parameters as well as environmental conditions, while eliminating confounding factors due to interspecies differences in pathophysiological pathways. Such systems facilitate the discovery of disease mechanisms and the establishment of causal relationships [5]. Furthermore, the creation of controlled micro-environments not only allows for accurate control over size, shape and form but for the mass-production of constructs suitable for screening, industry and clinical applications as well [6].

Various cellular populations are often used to derive disease models, ranging from differentiated cells to various stem and progenitor groups [7]. Though embryoid bodies (EB), stem cell-based models of embryos (SCME) and organoids are all three-dimensional (3D) cellular aggregations, they represent different organizational and developmental states. While EBs and SCMEs usually correspond to immature tissue states, organoids recapitulate more mature compositions. In the case of cardiac systems, however, a wide array of different terminologies is used to describe tissue models aimed at recapitulating embryonic or fetal tissues [8,9]. Recent advancements in developmental research and biotechnology have allowed for the construction of various models of embryonic development as well as organoids resembling embryonic and adult cardiac tissue. These models can be used to effectively mimic many aspects of congenital and genetic disease along with any associated tissue injury. The purpose of this review is to thus analyze and review the stem cell types used to generate EB, SCME and organoids as well as summarize and present the various cardiac systems that can be generated to recapitulate immature embryonic/fetal cardiac tissues suitable for disease modeling.

## 2. Stem Cells for Cardiac Disease Modeling

Cardiovascular disease can include many different disease presentations, including coronary artery disease (CAD) [10], cerebrovascular disease [11,12], peripheral artery disease (PAD) [13], aortic disease [14,15,16,17,18] as well as disorders affecting the myocardium [19], pericardium [20], cardiac valves [21,22], rhythm [23] and cardiac function [24,25]. The associated phenotypes usually range from derangements appearing during development [26,27,28,29,30,31], genetic disorders with a monogenic [32,33] or polygenic component [34,35], disorders affected by environmental factors [36], complex disease processes [9,37,38] as well as disorders arising due to effects of pharmacological compounds [39,40].

Disease models can be generally classified into systems utilizing living organisms (in vivo modeling), components of a living organism (ex vivo modeling) and cellular systems (in vitro modeling) ranging from single-cell and two-dimensional (2D) cultures [41] to 3D constructs [42,43] and organ-on-chip paradigms [2,44,45]. In silico models, i.e., assays that make use of and analyze varying amounts of data via computational modeling, have also been developed [46]. Despite the simplicity and cost-effectiveness of 2D cultures as well as their widespread use throughout preclinical research, they often cannot appropriately recapitulate the structural nuances of native tissue without additional modifications. Examples of such modifications can include the sandwich method comprising multiple, stacked Matrigel layers [47] and surface micropatterning [48,49]. Cardiac tissue is a complex 3D structure, composed of many different cell types interacting with one another as well as with elements of the extracellular matrix (ECM), characterized by specific biomechanical properties [50]. This highlights the need for more complex and efficient 3D environments to recreate it [7,51].

EBs and associated embryonic development derivatives, along with the organoid models discussed in greater detail in later sections, are all 3D culturing systems. They generally comprise self-aggregating and self-organizing cellular systems maintained under suspension conditions. These models have facilitated the study of morphogenesis, tissue development, structure/function and disease affecting embryonic/fetal and adult tissues [8,38,52]. However, they can often lack perfusion, innervation and resident immune cell populations, necessitating the separate addition of such characteristics [53,54,55,56].

### Stem Cells: Definition, Classification and Disease Modeling

Stem cells are generally defined as non-differentiated cells characterized by their ability to self-renew and generate differentiated daughters; they can be classified into multiple different subtypes based on differentiation potential, ranging from totipotent populations to various pluripotent and multipotent cell groups. Totipotency generally characterizes the developmental period during which widespread genomic activation occurs, known as embryonic genome activation (EGA) or zygotic genome activation (ZGA) [57,58]. This period occurs around the 2-cell (2C) stage in murine and 4-cell (4C) stage in human embryos [59,60], although other studies place such events earlier [61]. These totipotent cell groups will eventually, through continuous cellular divisions, generate more differentiated cells from all intraembryonic and extraembryonic germ layers [62,63,64,65].

Pluripotent stem cells (PSC) can generate cells and tissues derived from intraembryonic germ cell layers (endoderm, mesoderm and ectoderm), although they are also capable of differentiating into various extraembryonic tissues as well, including hypoblast [66], extra-embryonic (primitive) endoderm [67,68,69] and extraembryonic mesoderm [70]. PSCs can be derived from the inner cell mass (ICM) and are thus termed embryonic stem cells (ESC), though human ESCs have been associated with ethical controversy due to their derivation from embryonic blastocysts [71]. PSCs can also be generated from various somatic [72] and stem/progenitor populations [73,74,75] through the use of specific transcription factors (TF). More specifically, TFs used for the reprogramming of somatic cells can include combinations such as OSKM, comprising Octamer-binding transcription factor 4 (Oct4), SRY (Sex-determining region Y)-box 2 (Sox2), Krüppel-like factor 4 (Klf4) and cellular Myelomatosis oncogene (c-Myc)) [76] and OSNL composed of Oct4, Sox2, Nanog and Lin28 [77,78,79,80].

The advent and progress of stem cell research has facilitated stem cell use in many aspects of research, including disease modeling. Various stem cell types can be utilized for the construction of 3D disease models [81,82] including cells with totipotent capabilities, such as extended pluripotent potential stem cells (EPSCs) used to derive blastocyst models (blastoids) [83]. These can be further combined with other stem cell types capable of extraembryonic lineage derivation, i.e., trophoblast stem cells (TSC), to generate EPSC/TSC blastoid models [83,84]. Chemically induced totipotent cells have also been used to generate EBs [85] and blastoids [64], eventually forming both embryonic and extraembryonic lineages. Successful recapitulation of embryonic development in these constructs can make models derived from such cells appropriate candidates for production of cardiac systems as well [86]. PSCs have also been commonly used to create embryoid and organoid models of disease [87], including cardiac models [6,8,9,38,53,88,89,90,91,92,93,94,95,96,97,98,99,100,101,102,103], perhaps due to their ability to produce differentiated cell types from all three embryonic germ layers as well as their ease of derivation, at least comparably to totipotent groups [64].

PSC are commonly used for the creation of EBs [104,105] and various other SCMEs [106,107,108,109], though in the case of organoids, other types, including adult multipotent stem cells, can be used as well. Different multipotent cell types can be thus applied, each leading to derivation of organoids corresponding to the organ/tissue of origin. Some examples of tissue/organ-specific organoids include hematopoietic organoids derived from hematopoietic stem cells (HSC) [110], intestinal organoids derived from intestinal stem cells isolated from intestinal crypts [111], adipose tissue [112], mammary gland [113,114], fallopian tube [115], taste bud [116], lung alveoli [117], salivary gland [118], esophagus [119] and thyroid organoids [120]. However, there are some drawbacks to the use of multipotent stem cells, some of which pertain to their limited differentiation capacity as well as difficulties related to harvest of the various adult stem cell populations [87]. In addition, with regards to the isolation of cardiac stem/progenitor cells, their status has often been contested while their isolation has proven difficult in adult organisms, as they are found in greater numbers during cardiac development [121].

## 3. Embryoid Bodies, Organoids and Associated Models

### 3.1. Stem Cell-Based Models of Embryos

EBs have been historically described as embryonic carcinoma cell conglomerations, observed to form aggregations similar to those found during early embryonic development [122]. In general, EBs are defined as 3D disorganized aggregations of ESCs, although they can self-organize under appropriate conditions, thus mimicking early developmental stages [52,123]. They are also utilized as pluripotency assays and in various PSC expansion/differentiation protocols [124,125], as they can be induced to form all embryonic germ layers (endoderm, mesoderm, ectoderm) [126]. Through further manipulation of signaling pathways additional 3D aggregations corresponding to early developmental stages can be generated [107,127], derived not only from ESCs but other PSC types as well. These constructs are usually named after the specific developmental stage they resemble, although some authors propose terms such as ‘embryoid’, ‘stem cell-based models of embryos’ or nomenclature based on culture characteristics to collectively refer to such aggregates [82].

Blastoids are generally characterized as SCMEs resembling pre-implantation blastocysts [128,129] composed of cellular groups from all expected cell lineages during this stage of development, including the epiblast, hypoblast and trophectoderm (TE). Blastoids are produced from a number of sources, pluripotent as well as totipotent, including ESCs or EPSCs alone [83,128], ESC combinations with trophoblast stem cells (TSCs) [130], TSCs with extraembryonic endoderm stem cells (XEN) [131,132] and EPSCs with TSCs [84]. Blastoids can also be generated from starter populations of somatic cells subjected to TF combinations known to induce pluripotency [80] in an appropriate blastoid growth medium (induced blastoid models-iBlastoids) [129,133,134,135].

Gastruloids, on the other hand, are SCMEs resembling gastrulation (peri-implantation developmental period) [136] and after appropriate lineage modification they can also be used for the development of cardiac models [101]. They are generally characterized by a break in structural symmetry, anteroposterior axial organization and elongation, as well as specification of three main embryonic germ cell lineages (endoderm, mesoderm, ectoderm) [107,137]. Additionally, they can exhibit areas resembling primitive streak (PS) progenitor groups in the posterior pole [138] along with groups corresponding to endoderm [139], cardiac mesoderm (anterior) and paraxial mesoderm (middle), the latter of which is often seen undergoing somitogenesis [140,141].

**Figure 1:**
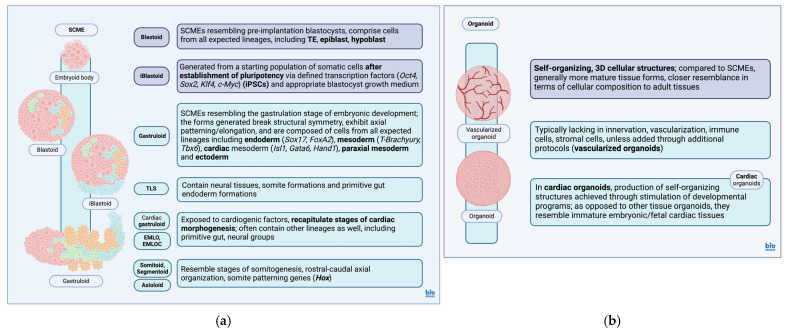
Summary of stem cell-based models of embryos (SCME) and organoids used for the modeling of disease. (**a**) SCME models can generally include blastoids and various gastruloids, while SCMEs mimicking key cardiac developmental stages via exposure to cardiogenic factors can be constructed as well. (**b**) Cardiac organoids are often generated as self-organizing 3D constructs recapitulating the development programs governing cardiac development (Created in BioRender.com). [54,82,96,102,106,133,136,152,158,165,166,167,168,172,173,174]. SCME, Stem cell-based models of embryos; TE, Trophectoderm; Oct4, Octamer-binding transcription factor 4; Sox2, SRY (Sex determining region Y)-box 2; Klf4, Krüppel-like factor 4; c-Myc, cellular Myelomatosis oncogene; iBlastoid, induced Blastoid; Sox17, SRY (Sex determining region Y)-box 17; FoxA2, Forkhead box protein A2; Tbx6, T-box transcription factor 6; Isl1, Insulin gene enhancer protein Islet-1; Gata6, Transcription factor GATA-6; Hand1, Heart- and neural crest derivatives-expressed protein 1; TLS, Trunk-like structures; EMLO, Elongating multilineage organized gastruloids; EMLOC, Elongating multilineage organized gastruloids with cardiogenesis; Hox, Homeobox protein. (**a**). Created in BioRender. Stougiannou, T. (2024) https://BioRender.com/q51d719 (accessed on 16 November 2024); (**b**). Created in BioRender. Stougiannou, T. (2024) https://BioRender.com/g01k583 (accessed on 16 November 2024).

Additional SCMEs have been generated as well, including trunk-like structures (TLS) exhibiting neural tube, somite and primitive gut formations [108,142], cardiac gastruloids produced via addition of cardiogenic factors [101] and gastruloids exhibiting co-developing cardiac/neural lineages along with primitive endoderm formations [102]. Gastruloid structures resembling various stages of somitogenesis (somitoids, axioloids, segmentoids) with anteroposterior patterning characterized by Homeobox (*Hox*) gene expression [143,144] have also been generated. As with most SCMES, manipulation of culture conditions can be applied to drive differentiation towards specific lineages [145] (Figure 1a).

### 3.2. Organoids

Organoids, as opposed to the various SCMEs, are 3D self-organizing structures [146] resembling more mature tissue architecture [147] and often described as structures that recapitulate tissue composition found in adult tissues [113,114,119]. With regards to cardiac systems, however, the term ‘organoid’ has been commonly used to describe embryonic and immature stages derived via recapitulation of embryonic developmental programs [8,9,38,92,98,99]. Cardiac microtissues, on the other hand, are derived from direct external assembly of appropriate cell types such as cardiomyocytes, endothelial cells, pericytes and cardiac fibroblasts [125,148,149,150], while engineered cardiac tissues also involve assembly of all these relevant cell types within an appropriate extracellular scaffold [151]. These, while useful for modeling mature cardiac tissues [150,151], cannot adequately recapitulate processes associated with development nor can they adequately recreate processes that result in the formation of cardiac defects, aspects for which constructs resembling embryonic states (cardiac organoids) are better suited [152].

Organoids can be derived from pluripotent, multipotent, as well as local progenitor populations isolated from specific tissues/organs [111,112,119,120]. They usually undergo periods of aggregation, proliferation, migration and eventually, differentiation into the appropriate cell groups [146]. This process can further involve transformation from a starting 2D culture to 3D culturing conditions [91,92,146,153]. Despite advances in organoid derivation and production as well as their capacity to mimic many of the nuances of 3D arranged tissues, they can sometimes lack key characteristics including mesenchymal elements, innervation, vascularization [154], as well as resident immune cells [125] and bacterial flora [155,156]. Furthermore, organoid tissue maturity compared to native tissues can be often lacking as well, although organoids themselves present a more mature stage of tissue composition compared to SCMEs, at least with regards to most organ/tissue organoids [38,82,147,156,157] (Table 1) (Figure 1b).

**Table 1:** Definitions of EBs, SCME groups and organoids. Ref, Reference; EB, Embryoid body; 3D, Three-dimensional; ESC, Embryonic stem cells; PSC, Pluripotent stem cells; SCME, Stem cell-based models of embryos; TE, Trophectoderm; Oct4, Octamer-binding transcription factor 4; Sox2, (Sex determining region Y) box 2; Klf4, Krüppel-like factor 4; c-Myc, cellular Myelomatosis oncogene; Sox17, (Sex determining region Y) box 17; PS, Primitive streak; FoxA2, Forkhead box A2; Tbx6, T-box transcription factor 6; Isl1, Insulin gene enhancer protein Islet-1; Gata6, GATA Binding protein 6; Hand1, Heart and Neural crest derivatives expressed 1; CHIR99021, Chiron; iPSC, induced Pluripotent stem cells; PSC, Pluripotent stem cells; BMP4, Bone morphogenetic protein 4; TLS, Trunk-like structures; EMLOC, Elongating Multilineage Organized Gastruloid with Cardiogenesis; Hox, Homeobox protein.

Model	Description	Ref.
EB	Disorganized 3D ESC aggregations, can organize into early embryonic structures, used as assays of pluripotency/first step during PSC expansion and differentiation protocols, capable of generating both intraembryonic (endoderm, mesoderm and ectoderm) and extraembryonic lineages.	[158,159,160,161]
Blastoid	SCMEs resembling pre-implantation blastocysts, derived from totipotent/pluripotent starter cell populations, comprising cells from all expected lineages at this stage of development (TE, epiblast, hypoblast).	[106]
	iBlastoid: generated from starting populations of somatic cells after reprogramming/establishment of pluripotency via defined transcription factors (*Oct4*, *Sox2*, *Klf4*, *c-Myc*) within the blastocyst growth medium.	[133]
Gastruloid	SCMEs resembling the gastrulation stage of embryonic development, exhibit characteristics found during this stage (breaking of symmetry, axial patterning, three major body axes, anteroposterior axial elongation, PS formation).	[107,136,143]
	Comprises cells from all expected lineages at this stage of development, including endoderm (*Sox17*, *FoxA2*), mesoderm (*Brachyury*, *Tbx6*), cardiac mesoderm (*Isl1*, *Gata6*, *Hand1*), paraxial mesoderm, ectoderm.	[140,162,163]
	Cardiac mesoderm, cranial lineage derivatives often underrepresented in general gastruloid models derived via CHIR99021-mediated Wnt signaling stimulation.	[136]
	TLS: gastruloids composed of neural tissues, somite formations surrounding a primitive neural tube and primitive gut endoderm formations, resemble the ‘trunk’ area of a developing embryo.	[108,164]
	Cardiac gastruloids, EMLOC gastruloids: gastruloids additionally exposed to cardiogenic factors, recapitulate stages of cardiac morphogenesis along with other lineages (multilineage cardiac-neural gastruloids).	[101,102,165]
	Somitoids, Axioloids, Segmentoids: gastruloids that recapitulate stages of embryonic somitogenesis with rostral–caudal axial organization, segmentation, expression of genes associated with somitogenesis and anteroposterior somite patterning (*Hox*).	[143,166,167,168]
Organoids	Self-organizing, 3D cellular structures, more mature tissue forms (cellular composition, tissue architecture) compared to EBs/SCMEs, cardiac organoids resemble more immature embryonic/fetal tissue forms (compared to other tissue organoids), generally lack innervation, vascularization, immune cells, stromal cells and bacterial flora (can be added separately).	[54,56,110,154,169,170,171,172,173]

## 4. Cardiac Models

### 4.1. Cardiac Models and Signaling Pathways

Cardiac morphogenesis commences during gastrulation when pluripotent epiblast cells traverse the anterior and middle PS at different times, generating different cardiac mesoderm subpopulations [96]. The first subpopulation to arise through this anterior migration is the first heart field (FHF) [96], typically extending over the embryonic midline and forming a crescent. FHF gives rise to left ventricular (LV) cardiomyocytes and some atrial populations. The second population to migrate anteriorly, the second heart field (SHF), produces right ventricular outflow tract (RVOT) cells and the remainder of the atrial groups [175]. These groups eventually arrange into the cardiac crescents. While both FHF and SHF groups give rise to endocardial progenitors, epicardial progenitors typically develop from a separate mesodermal lineage, the pro-epicardium [176]. Signaling from the anterior foregut endoderm (AFE) along with general folding of the embryo further facilitates cardiac development [177,178,179]. Additional lineages also incorporate into cardiac tissue until maturity including cardiac neural crest cells (cNCC), neuronal groups and immune cells, all interacting with each other as well as with aspects of the ECM, further influencing cell and tissue specification [95,96,180].

Most protocols involve the initial induction of mesendoderm (T or Brachury) via stimulation of the Wnt signaling pathway, achieved through the use of small molecules such as CHIR99021 (CHIR, Chiron) and 6-bromoindirubin-3-oxim (BIO) [181]. CHIR99021 is an inhibitor of glycogen synthase 3β (GSK3β) and thus a Wingless-related integration site (Wnt) pathway agonist [182]. Via stimulation of the canonical Wnt pathway, β-catenin phosphorylation and degradation by the GSK3β complex is prevented, leading to the transcription of various downstream targets [183]. Though short-term GSK3β inhibition maintains pluripotency, long-term inhibition usually leads to upregulation of endoderm and mesoderm genes [145,184,185,186] including cardiac mesoderm [182]. In response to CHIR99021-mediated Wnt pathway stimulation, symmetry-breaking events are induced, including axial elongation and anteroposterior patterning [145]. These events are mediated through cell–cell adhesion remodeling and are usually characterized by an increase in the expression of T or Brachury and Snail. Snail upregulation, in particular, is associated with downregulation of epithelial cell fates and stimulation of epithelial-to-mesenchymal (EMT) transition events [184], marking the beginning of gastrulation. CHIR99021 administration along with the resulting increase in BMP4 expression simulates endogenous BMP4/Nodal/Wnt signaling, which is in turn associated with the development of posterior embryo structures, downregulation of genetic markers associated with anterior embryo structures and inhibition of neural lineages [184,187].

**Figure 2:**
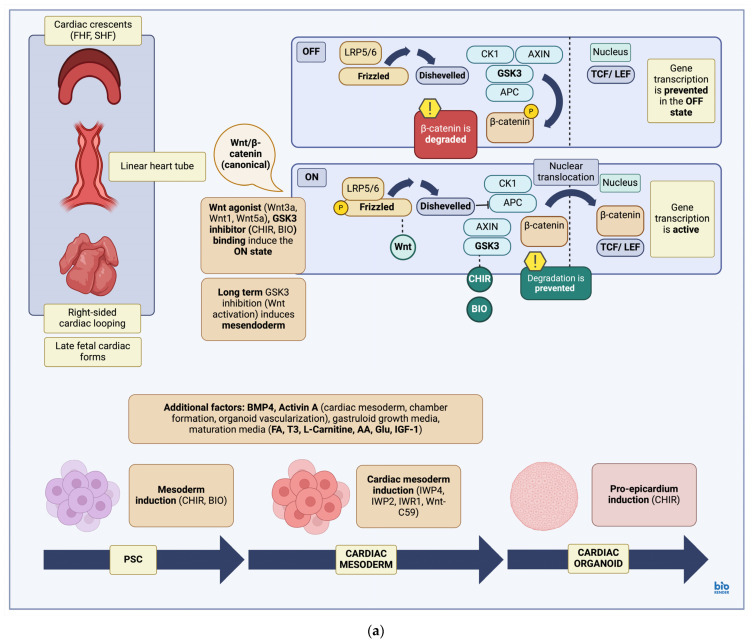

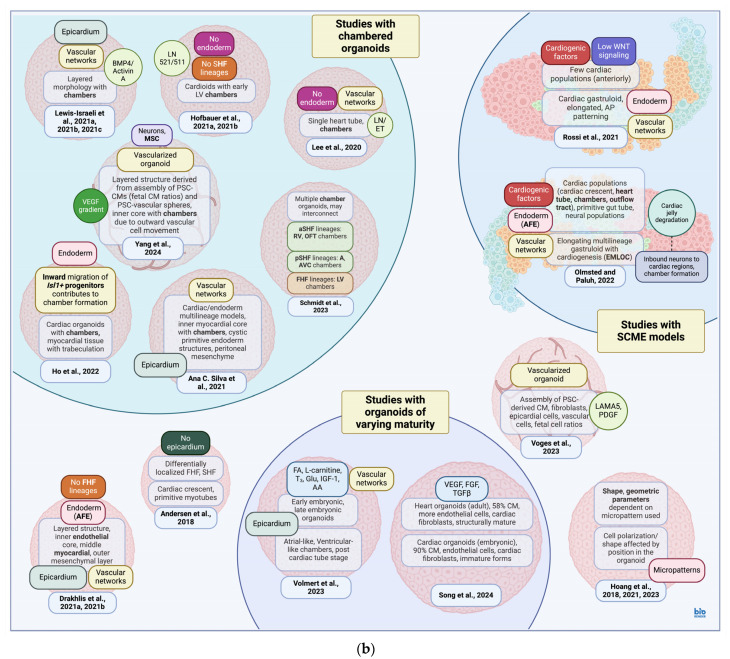
Key characteristics of models used to recreate cardiac systems in vitro and relevant signaling pathways. (**a**) The canonical Wnt signaling pathway involves stimulation of the LRP5/6-Frizzled receptor by Wnt ligands, allowing for the destruction complex (CK1, AXIN, GSK3β, APC) to interact with Frizzled and thus lose its ability to degrade β-catenin. β-catenin is then free to translocate to the nucleus and form complexes with TCF/LEF, inducing transcription of various genes. The pathway can be similarly activated by small molecule inhibitors of GSK3β which prevent β-catenin degradation, allowing for its nuclear translocation. In the absence of LRP5/6-Frizzled stimulation, the destruction complex (CK1, AXIN, GSK3β, APC) binds and targets β-catenin for degradation. Protocols used for cardiac organoid derivation usually entail initial activation of Wnt signaling with small molecules (CHIR, BIO) that inhibit GSK3β, followed by Wnt signaling inhibition to induce cardiac mesoderm formation. In some studies, a third wave of Wnt signaling stimulation can be applied to induce pro-epicardium formation and increase complexity of the derived construct. Additional factors such as BMP4 and Activin A can be used to facilitate cardiac mesoderm formation and special media can be used to enhance organoid maturation by recapitulating late metabolic and hormonal in vivo gestation conditions. Gastruloids are usually generated through use of appropriate gastruloid growth medium along with additional factors depending on the sought-after lineages. Cardiac tissue morphogenesis generally comprises the initial formation of cardiac crescents, followed by migration of cardiac progenitors towards the midline and formation of a single heart tube. Eventually, chamber formation, right-sided looping and tissue growth/maturation occurs, generating late fetal cardiac forms arranging into the well-known cardiac shape found in postnatal and adult organisms. (**b**) Various models can be produced to recreate cardiac systems in vitro: some exhibit chamber formations (shown in the top left area), others are derived with protocols aimed at inducing structural/functional maturation (shown in the bottom area) while others are developed as cardiac gastruloid models (shown in the top right area). The characteristics of each system, along with any additional factors that induce special conditions; for example, the addition of LN/ET or LN521/511 to aid in chamber formation in some models, are noted as well. While many of these organoids exhibit vascular networks, others are derived with protocols specifically aimed at inducing vascularization (constructs noted as ‘vascularized organoids’). Organoid constructs not fitting in any of those three categories are pictured outside of the three circular boundaries (created in BioRender.com) [8,9,38,53,88,89,90,91,92,93,94,95,96,97,98,99,100,101,102,103,196,250]. Wnt, Wingless related integration site; LRP5/6, Low density lipoprotein-related receptors 5/6; CK1, Casein kinase 1; GSK3, Glycogen synthase kinase 3 beta; FA, Fatty acid; APC, Adenomatous polyposis coli; CHIR, CHIR99021 (Chiron); BIO, BIO, 6-bromoindirubin-3-oxim; IWP2, Inhibitor Wnt production-2; IWP4, Inhibitor Wnt production-4; IWR1, Inhibitor of the Wnt response-1; AXIN, Axin-1; Wnt3a, Protein Wnt-5A; Wnt5a, Protein Wnt-5A; Wnt-C59, Wnt protein C59; TCF/LEF, T-cell factor/lymphoid enhancer factor; LN, Laminin N-terminal; ET, Entactin; FGF, Fibroblast growth factor; BMP, Bone morphogenetic protein; BMP4, Bone morphogenetic protein 4; FHF, First heart field; SHF, Second heart field; PSC, Pluripotent stem cells; hPSC, human Pluripotent stem cells; AFE, Anterior foregut endoderm; FGF2, Fibroblast growth factor 2; RA, Retinoic acid; VEGF, Vascular endothelial growth factor; Wnt, Wingless-related integration site; aSHF, anterior Second heart field; pSHF, posterior Second heart field; RV, Right ventricle; OFT, Outflow tract; AVC, Atrioventricular canal; LV, Left ventricle; IGF-1, Insulin-like growth factor 1; SCME, Stem cell-based models of embryos; AP, Anteroposterior; bFGF, beta Fibroblast growth factor; AA, Ascorbic acid; TGFβ, Transforming growth factor beta; TGFβi, Transforming growth factor beta inhibitor; EMLOC, Elongating multilineage organoid with cardiogenesis; CM, Cardiomyocyte; Glu, Glucose; Isl1, Insulin gene enhancer protein Islet-1; MSC, Mesenchymal stem cells. (**a**). Created in BioRender. Stougiannou, T. (2025) https://BioRender.com/r28q564 (accessed on 16 November 2024); (**b**). Created in BioRender. Stougiannou, T. (2025) https://BioRender.com/p43e753 (accessed on 16 November 2024).

Cardiac mesoderm is then induced, usually through Wnt pathway inhibition via various molecules such as Inhibitor Wnt production-4 (IWP4) [188], IWP2 [189], inhibitor of the Wnt response-1 (IWR1), a tankyrase inhibitor [190] and Wnt-C59, an inhibitor of Protein-serine O-palmitoleoyltransferase porcupine (PORCN) [191,192]. Modification of timing and dosage of the various GSK3β and Wnt inhibitors used, as well as supplementation with additional molecules such as insulin to improve cardiomyocyte survival rate, can further improve differentiation efficiency [193,194]. In addition, application of retinoic acid (RA) facilitates anteroposterior patterning in the lateral plate mesoderm [195], in turn aiding in the anteroposterior specification of the second heart field (SHF). In some studies, an additional cycle of CHIR99021-mediated Wnt activation can be applied after cardiac mesoderm specification, allowing for the derivation of epicardial lineages [9,196]. Within cardiac mesoderm FHF and SHF lineages can be then specified [89,90]. While anterior SHF lineages (aSHF) contribute to outflow tract (OFT) and right ventricle (RV) formation, posterior SHF lineages (pSHF) contribute to atria and the sinus venosus [88,197] (Figure 2a).

### 4.2. Cardiac Models: Production and Composition

Pluripotent cell aggregations have been stimulated to self-organize into the many structures observed during cardiac morphogenesis. EBs can be developed using various signaling factors, including fibroblast growth factor 4 (FGF4), bone morphogenetic protein 4 (BMP4) [198], leukemia inhibitory factor (LIF) as well as BIO, into more complex heart organoids [89]. Additional supplementation with laminin (LN)/Entactin (ET) complexes containing laminin-111 (α1β1γ1) facilitates chamber development. Cardiac organoids produced in this manner exhibit an average size of 835.1 μm, with cardiac crescents eventually merging into a single heart tube which undergoes looping and chamber formation. Both FHF and SHF cardiac progenitors have been derived with this protocol, with 60% of all organoids exhibiting beating activity (rhythmic Ca^2+^ transient peaks) on day 3, while this increases to around 80% on days 5–6 [89].

FGF signaling factors are important for organoid development as well as chamber derivation. More specifically, FGF4 is associated with the highest efficiency of chambered organoid generation, reaching a maximum of 88%, compared to FGF10 which is associated with a maximum efficiency of 42%. Derivation efficiency associated with use of basic FGF (bFGF or FGF2), on the other hand, is 58%. These differences can be explained by the higher affinity of bFGF and FGF4 for FGF receptor 1 (FGFR1) [199,200]. Regarding morphogenetic aspects, heart tube and chamber formation occur only with use of LN/ET and not Matrigel, as the latter is associated with chambered organoid derivation efficiency of only 8%. Moreover, inhibition of FGFR1 signaling prevents organoid development altogether [89], highlighting the effects of FGF and LN/ET on chamber formation and organoid development. Though most cardiac cell types are observed including FHF/SHF progenitors, atrial- and ventricular-like cardiomyocytes, Purkinje cells, endothelial cells and conglomerations resembling axon formations (due to BMP4 culturing), no endodermal lineages have been derived with this protocol [89].

Often, cardiac organoids can be derived to contain differentially localized cardiomyocyte progenitors via protocols utilizing CHIR99021 (mesoderm) and Activin/BMP4 (cardiac mesoderm) [198,201,202]. Use of BMP4 has a more potent cardiogenic effect compared to Activin A, as a 0.75 ng/mL increase in BMP4 concentration produces a 10-fold (27%) increase in mean cardiomyocyte numbers, whilst a similar increase in Activin A concentration produces a much smaller increase. Regarding cardiac morphogenesis, the organoids exhibit cardiac crescents and primitive myotube formations composed of FHF and dorsally/posteriorly located SHF progenitors. FHF and SHF progenitors form distinct groups within the constructs, both of which appear at the same time (~120–140 h) in culture, though their precursors are specified earlier. BMP is important for cardiac development in the organoid, as BMP signaling leads to the upregulation of an inhibitor of DNA binding (Id) 1 which facilitates the induction of FHF populations [203,204]. FHF specification is thus induced via BMP/Smad signaling. SHF specification, on the other hand, is dependent upon cooperation of BMP4 with endogenous inducers of canonical Wnt signaling and thus a Smad-independent BMP/Wnt signaling pathway, as administration of Wnt inhibitors abolishes any effect BMP4 has on SHF specification [90,205]. SHF populations exhibit higher rates of proliferation compared to FHF populations in this model, owing to the increased expression of negative cell cycle regulator proteins in the latter. This eventually leads to a larger SHF population (almost doubling in number within 36 h in culture) in comparison to FHF. No epicardial groups have been derived in this study [90].

Specific shapes in cardiac organoids can be achieved via an initial 2D induction stage (CHIR99021, IWP4) in micropatterned plates, allowing for PSC aggregation into a 3D form [92,93]. The organoids exhibit a central area composed of cardiac tissue with some differentiated cardiomyocytes (cardiac Troponin T-cTnT), surrounded by an area of smooth muscle-like stromal cells (Transgelin/Smooth muscle protein 22-Tagln/Sm22). Shape and size can vary depending on the micropattern employed, as evidenced by the observed variations in area ratio, height and full width at half-maximum (FWHM). Small micropattern shapes (200 μm) are associated with low production efficiency (20%) compared to all other sizes (~80%), although they produce organoids with the largest area ratio of cardiac tissue, at ~0.8, compared to all others ranging between 0.4 and 0.8 instead. Better 3D morphology, on the other hand, characterized by higher median values for height (~200 μm) and FWHM (~400 μm), is usually associated with larger patterns (600 μm). Pattern size affects functional parameters as well, with smaller patterns producing organoids with higher contraction and relaxation velocities along with greater prolongation in contraction duration. In turn, this predisposes constructs derived from smaller patterns to arrhythmia and abnormal diastolic function. Higher beating rates, on the other hand, are associated with larger patterns. The shape of the initial micropattern used can also affect organoid characteristics and functional parameters. Various micropattern shapes can be used (circle, triangle, square) to derive organoids, although circle shapes are associated with higher median values for area ratio (~0.6), height (~250 μm) and FWHM (~300 μm). Shape also affects functional characteristics such as contractile function and conduction velocity, with circular shapes producing the best overall results [92].

Quadrilateral and pentagram pattern formations can also be employed to generate organoids, with geometrical confinement in each pattern influencing stem cell differentiation. This is usually due to effects on cell positioning, diffusible signals [206] and actomyosin remodeling/contractility [207] with factors such as aspect ratio and central area affecting organoid structure and function. With quadrilateral shapes, increases in the aspect ratio negatively affect construct self-assembly and physical parameters, although elongated patterns are generally associated with improved contractile function [91]. Increasing aspect ratio is thus associated with decreasing organoid height. Regarding pentagram shapes, on the other hand, an increase in the central area is associated with an increase in median organoid height values by ~50 μm, while ideal star shape patterns are associated with the greatest height range (maximum height values of almost 300 μm). Geometrical parameters affect contractile function as well, leading to a decrease in median beating rate by ~20 bpm with increasing aspect ratio and progressive increase in contraction/relaxation velocity, at least in quadrilateral shapes. For pentagram shapes, on the other hand, contractile function exhibits variation; maximum contraction and relaxation velocities, both at ~20 μm/s, are observed with ideal star shapes [91].

The polarization and shape of each individual cell is relevant to its position within the construct, with peripherally situated cells exhibiting elongated polarized shapes, compared to centrally located cells exhibiting round shapes. This further highlights the effect of geometric parameters on organoid structure, brought about due to variation in the biomechanical parameters and as a result, biological pathways associated with mechanotransduction [91]. Smaller patterns, as well as patterns that are associated with more cells located peripherally, generally lead to higher mechanical stress in cells at the perimeter of the structure. This leads to stimulation of Rhoa/ROCK signaling and activation of downstream effectors such as Yes-associated protein (YAP)/Transcriptional co-activator with PDZ binding motif (TAZ). YAP/TAZ functions as a mechanotransducing complex, responding to changes in cell polarity, surface area and density. Cells at the periphery are associated with polarized, elongated shapes, while cells with more rounded morphology are found near the center. These rounded cells exhibit fewer intercellular adhesions and lower YAP/TAZ activity, in turn affecting gradients of patterning signals and eventually, organoid formation [91,208,209].

Immature heart-forming organoids (HFO) can be generated after encapsulation of starter hPSC populations in Matrigel and sequential mesoderm (CHIR99021)/cardiac mesoderm (IWP2) induction. Constructs generated in this manner exhibit a layered and overall spherical morphology, while the protocol is associated with an organoid formation efficiency of 88% [94]. The layers of the organoids generated with these steps comprise an inner core (IC) composed of endothelial progenitor groups (~4% of total population) coalescing into primitive vascular networks and an outer myocardial layer (ML) composed of SHF cardiac progenitors and differentiated atrial/ventricular cardiomyocyte groups. Regarding cardiac progenitors, while SHF cells comprise ~77% of the total population on day 7, this percentage drops to ~25% on day 13, highlighting the progressive differentiation of cardiomyocytes in the construct. However, no FHF lineages, based on detection of the marker *T-box transcription factor TBX5* (*Tbx5*), have been identified. Cardiomyocytes generated with this protocol are mostly of the ventricular phenotype (75.5%) characterized by low upstroke velocity, with only a small subset exhibiting atrial-like characteristics (5.7%). The outermost layer of the organoids is composed of a mixture of *homeobox protein Nkx2.5* (*Nkx2.5*)*+* and (*Nkx2.5*)− cardiac progenitor lineages, mesenchymal, primitive septum transversum (ST) and proepicardial groups [8]. Crosstalk between AFE (~22% of total cell population) and cardiac mesoderm further contributes to the development of cardiac lineages (including endocardium) in this model, as evidenced by the presence of an endocardial layer between the endoderm and cardiac mesoderm [8,94,210,211,212].

Often, cardioids can be engineered with cavities forming as part of their structure [88,95,96]. In some studies, this is achieved via additional administration of fibroblast growth factor 2 (FGF2) [213], bone morphogenetic protein 4 (BMP4)/Activin A [198,214], IWP2, CHIR99021, insulin and RA [95,96]. Protocols that utilize intermediate CHIR99021 and high Activin A concentrations generally produce higher levels of atrial marker expression, compared to low CHIR99021/Activin A concentrations associated with higher expression of ventricular markers instead. The chambered cardioids generated are thus of mixed atrial and ventricular phenotype, originating from FHF lineages. Variations in the concentrations of CHIR99021 used are also associated with variations in the diameter of the final constructs, as an increase in CHIR99021 concentration from 4 to 5 μM produces the greatest increase in mean diameter values, to ~800 μm from an initial value of 600 μm [95,96].

Chamber formation in these studies is facilitated by the presence of laminins 521/511. The process involves the initial accumulation of dense tissue (high F-actin, N-cadherin expression) around the periphery, leaving areas with lower tissue density (low N-cadherin expression) towards the center that will eventually hollow out and form cavities. Chamber development is further dependent on high levels of Wnt/BMP4 signaling early during mesoderm specification, as chambers start to form between days 1.5 and 2.5 in culture, between the end of the mesoderm induction period and the start of the cardiac mesoderm derivation. This is further corroborated by BMP inhibition with Noggin and LDN193189 during the early days of cardiac mesoderm induction. Although BMP inhibition has no effect on mean organoid cell numbers, it decreases mean cardioid diameter by ~220 (Noggin) and ~200 (LDN193189) μm while also preventing cardiac chamber expansion. Generation of cardioid chambers in this model thus entails BMP-mediated activation of gene targets such as *Heart- and neural crest derivatives-expressed protein 1* (*Hand1*), *Homeobox protein Nkx-2.5* (*Nkx2.5*) and *Iroquois-class homeodomain protein IRX-3* (*Irx3*) [95,96]. Cardiomyocyte gene expression has also been analyzed and compared between 3D cardioids, 2D cardiomyocyte cultures and hPSCs at various time points. Upregulation of most cardiomyocyte genes associated with key functional processes in 3D cardioids and cardiomyocytes assembled in 3D has been observed, compared to PSCs and cardiomyocytes in 2D cultures. Cardioids generated with this protocol generally assume an early left ventricular phenotype [96].

Human heart organoids (hHO) have also been produced from EBs, once again via mesoderm/cardiac mesoderm induction. Repeat administration of CHIR99021 can be further used after cardiac induction to induce formation of proepicardial tissues and increase tissue complexity [9,196]. Optimal CHIR99021 concentrations associated with the highest number of cardiomyocytes in thus study, as detected by *Tnnt2* expression, is 4 μM CHIR99021 for a resulting mean ratio of 64.9%. hHOs generated generally possess a layered morphology, with myocardial tissue comprising SHF/FHF progenitors, atrial and ventricular cardiomyocytes and relevant cardiac tissue types (endocardial, endothelial, epicardial and cardiac fibroblast groups). BMP4/Activin A signaling additionally facilitates formation of cardiac mesoderm, chambers and inter-chamber connectivity, while it also increases organoid growth by 15%. BMP4/Activin A administration further contributes to organoid vascularization, evident by an increase in *PECAM1+* endothelial cell numbers by 160%. The epicardial groups in this model (associated with pro-epicardium organ specification) are specified after a repeat wave of Wnt signaling activation (CHIR99021), once cardiac mesoderm specification is complete. Epicardial tissue is peripherally situated in the organoid (adjacent to myocardial layers) and contributes to organoid complexity, with 2 μM CHIR99021 generating the most biologically relevant cellular ratios of epicardial (10–20%) to cardiomyocyte groups (60–65%) [9,97,196]. Assessment of cardiomyocyte gene expression and functionality reveals upregulation of key genes associated with cardiac function as well as similarities with the fetal heart genetic expression. Genes evaluated include those associated with cardiac conduction, contractility, calcium handling pathways, maturation as well as oxidative phosphorylation. Furthermore, comparison of gene expression between organoid cardiomyocytes, monolayer cardiomyocytes and fetal heart tissue reveals more genes upregulated in organoid cardiomyocytes compared to monolayer cardiomyocytes) [9,97].

Chambered cardiac organoids (CCO) can also be generated from starter PSC populations via sequential mesoderm/cardiac mesoderm induction protocols [98,189]. Some studies specifically induce *Isl1+* cardiovascular progenitors (CVP), leading to the generation of spherical organoids composed of most cardiac tissue cell types (cardiomyocytes, vascular smooth muscle cells-VSMC, cardiac fibroblasts) along with endoderm populations. The chambers in these models are found within the organoid core and are surrounded by trabeculated myocardial tissue. Chamber formation in this model is attributed to the inward migration of *Isl1+* CVPs, an event dependent on Wnt/BMP4 signaling and the relative ratios of *Isl1+* progenitors grown in the presence of Endothelin-1 and expressing *signal-regulatory protein alpha* (*SIRPa*)/*Endothelin-1 receptor* (*SIRPa+ EDNRA+* CVPs) [215,216] and *SIRPa+ EDNRA−* cardiomyocytes. The highest proportion of contracting CCOs (~60%) can be created with compositions comprising equal amounts of *SIRPa+ EDNRA+* CVPs and *SIRPa+ EDNRA−* cardiomyocytes. On the other hand, sole use of *SIRPa+ EDNRA−* cardiomyocytes produces the lowest amount of contracting CCOs (less than 20%), while sole use of *SIRPa+ EDNRA+* CVPs produces contracting organoids with no chamber formations. Chambers generally form only after a minimum of *Isl1+* CVPs has accumulated [98]. With increasing days in culture, cardiomyocytes upregulate genes associated with structure, function and metabolism, including genes such as *Myosin light chain 7 (Myl7)*, *Myosin heavy chain 6 (Myh6)* and *Ryanodine receptor 2 (Ryr2).* Downregulation of progenitor genes such as *Isl1* and *Myocyte-specific enhancer factor 2C (Mef2c)* is also observed, highlighting the progressive maturation of CCO cardiomyocytes in culture [98].

Multiple chambered organoids can also be generated, each corresponding to different chambers within an embryonic heart. These are produced through induction of starter PSC aggregates towards mesoderm/cardiac mesoderm via Wnt inhibition and stimulation of Activin/Nodal signaling. Following induction of cardiac mesoderm, aSHF and pSHF progenitors are derived via inhibition of Wnt/Transforming growth factor beta (TGFβ) signaling, with additional supplementation of RA for pSHF groups [88]. RA inhibits pathways directing aSHF lineage differentiation, via upregulation of *T-box transcription factor 5 (Tbx5)* [88], while additional studies have shown an increase in *Wnt2* and *Homeobox protein Hox-B1* (*HoxB1*) transcripts after RA supplementation, supporting differentiation towards pSHF lineages [197]. FHF lineages, on the other hand, can be produced via inhibition of Wnt signaling and additional supplementation with BMP4, FGF and insulin [88,217,218]. FHF cardioids are generally larger, at ~2 million μm^2^, compared to aSHF- and pSHF-derived chambers, while FHF cell numbers are ~80,000, similar to pSHF-derived chambers and smaller than aSHF-derived groups, which are at ~150,000 cells (day 9.5). Cell size, on the other hand, generally remains the same across all cardioid types. The larger size of FHF organoids can be thus attributed to the increased extracellular material in these constructs. Chamber formation in FHF-derived constructs commences sooner as well, at around day 2, as opposed to day 3 for all other SHF-derived groups [88].

All these constructs often co-develop in close proximity, allowing for inter-chamber connections to form. FHF lineages give rise to left ventricle (LV) cardioids, aSHF lineages produce right ventricle (RV) and outflow tract (OFT) cardioids, and finally, pSHF lineages generate atrial and atrioventricular (AVC) canal organoids. Almost 100% of all LV cardioids exhibit spontaneous contractile function at day 6.5, though this percentage falls to less than 25% at day 9.5 and is associated with a decrease in *Potassium/sodium hyperpolarization-activated cyclic nucleotide-gated channel 4* (*Hcn4*) expression. A decrease in the percentage of spontaneously beating RV, OFT organoids from days 7.5 to 9.5 is also observed, although this decrease is smaller than the reduction observed in LV organoids, less than 20%. On the other hand, the percentage of atrial and AVC organoids exhibiting spontaneous contraction remains stable at ~100% throughout days 7.5–9.5 in culture and is associated with the highest levels of *Hcn4* expression [88]. These differences in spontaneous contractility and ion channel expression across different chambers and timepoints in development highlight the differences in the automaticity/action-potential (AP) characteristics often observed within the developing heart [88,219].

Organoids can be additionally modulated via manipulation of metabolic/hormonal parameters to mimic conditions observed during late in vivo gestation and induce maturation [99]. Early and late embryonic heart organoids are thus derived from starter EB aggregations via protocols aimed at Wnt pathway modulation. Early to late organoid transition, in particular, is stimulated by exposure to maturation media generally composed of fatty acids (FA), L-carnitine, T_3_ hormone (T3), glucose and Insulin-like growth factor-1 (IGF-1). Maturation media allow for the recapitulation of the normally occurring, metabolic transition from glucose to FA metabolism during late development [220]. Variations of maturation media have been thus developed, including maturation medium (MM) comprising basal medium along with FA, T3 and Carnitine, enhanced maturation medium 1 (EMM1) comprising MM along with glucose and ascorbate and enhanced maturation medium 2/1 (EMM2/1) comprising the sequential administration of EMM1/IGF-1, followed by repeat EMM1 administration. Despite the use of varying maturation media, total organoid area across different conditions exhibits a similar range of 0.5–1.0 mm^2^ (day 30). No differences between the control and experimental conditions are observed for spontaneous organoid contractility either, with almost all organoids exhibiting contractile activity. Regarding structural characteristics, however, EMM1 medium produces sarcomeres with the greatest mean length, at ~1.58 μm [99].

Maturation of these organoids is further evident by the upregulation of genes associated with mitochondrial respiration when EMM2/1 is applied. EMM2/1 medium conditions thus represent the parameters most closely recapitulating in vivo conditions of metabolic maturation. This event is also associated, by an increase in mitochondrial area/cell, with the highest fold-change observed for EMM2/1, at ~3.10. Organoids in this model are characterized by anteroposterior patterning, mediated via RA signaling gradients, specification of atrial and ventricular chamber formations [221] along with a proepicardial organ. Organoids grown in MM medium exhibit the highest percentage of atrial and ventricular cardiomyocytes, at 34% and 27%, respectively. The organoids also exhibit chamber formations comprising an upper and lower compartment. While lower chambers are composed of thicker walls composed mostly of *Tnnt2+* cardiomyocytes, upper chambers are less dense and composed of *Tnnt2+* cardiomyocytes along with *Wilms Tumor protein 1 (WT1)+* epicardial cells. Use of the EMM2/1 medium produces the greatest increase in chamber area for *Tnnt2+*/*WT1+* chambers, with a 1.54-fold and 1.98-fold increase, respectively [99].

In many of these organoids, derivation processes aim at specifically generating cardiac lineages, although many contain additional lineages such as endoderm [8] and neural populations [89]. During development, the morphogenesis of ventral structures (foregut invagination, head/cardiac localization) is regulated by factors such as Bone morphogenetic protein 2 (BMP2) secreted by the visceral endoderm and acting upon the developing epiblast [179]. Folding of the ventral endoderm facilitates formation of the midline-positioned linear heart tube, with cardiac groups initially co-migrating with converging endodermal groups, an event mediated by Sphingosine-1-phosphate (S1P) and G protein-coupled S1P receptor 2 (S1pr2) (S1pr2/Gα_13_) signaling [222,223]. Absence of S1pr2 or its cellular transporter *Spns2* usually results in cardia bifida in zebrafish models owing to defects in this migration [224,225,226]. Following this first phase of co-migration, myocardial subduction occurs with myocardial groups moving from dorsal to ventral relative to the converging endoderm (subduction) and assembling into two layers. While subduction does occur in the absence of S1pr2/Gα_13_ signaling, it lasts longer and is not as effective. Finally, the last migration phase, medial migration of myocardial precursors and formation of a heart cone, occurs independent of endoderm movements [223] with peripherally situated myocardial groups in anterior and posterior locations altering their direction and exhibiting angular movement towards endocardial progenitors. Specification of endocardial groups is also facilitated by signals from the anterior endoderm [210].

Not only is myocardial assembly into a midline cone facilitated by endoderm but it is further influenced by endocardial populations as well, which is evidenced by the emergence of dysmorphic cones in the absence of endocardium [227]. The endocardium generally originates from endothelial/hemopoietic progenitor groups [228] or according to other studies, from a *NFATc1+* common myocardial/endocardial progenitor [229,230]. Midline migration of the endocardium is also facilitated by the S1pr2/G_α13_-mediated endodermal convergence as experimental S1pr2/G_α13_ deficiency leads to endocardium malposition, thus preventing the medial migration of myocardial groups [231]. The endocardium generally contributes to the development of endocardial cushions, atrial/ventricular septae, valve leaflets, trabeculation during chamber development [232] and formation of the cardiac conduction system [233,234]. Epicardial cells (*WT1+*) are derived from the pro-epicardium organ, a transient structure appearing during cardiac development. The pro-epicardium is important for cardiac development as well, due to its contribution to cardiac fibroblast and coronary smooth muscle cell populations and the production of paracrine signals that facilitate maturation and compaction of myocardial tissue. It can be specified via protocols involving temporal modulation of RA, CHIR99021 and BMP4 availability [235] as well as protocols involving a repeat Wnt signaling stimulation step (CHIR99021) after cardiac mesoderm induction [9,97,196]. Finally, non-myocyte pacemaker cells comprising the cardiac conduction system have been usually derived with protocols involving BMP and RA-based signaling with associated downregulation of FGF signaling pathways, after mesoderm induction has occurred. Though usually associated with low differentiation efficiencies, these can be increased to 35% with modulation of BMP4 concentrations [236].

Development of organoid models with multiple co-developing lineage groups can facilitate cardiac development [8,211,212], evident in cardiac/endoderm co-lineage models. These are produced via sequential induction of mesendoderm/cardiac mesoderm (IWP2) as well as additional supplementation with ascorbic acid (AA) [100,237]. The organoids produced in this manner possess a spherical shape with cystic structures corresponding to endodermal lineages (day 30) and a central myocardial core comprising chamber and septae formations. Myocardial groups in these constructs arrange into atrial/ventricular compartments and are surrounded by mesenchymal tissues containing glycosaminoglycans and hyaluronic acid resembling peritoneal or sub-epicardial tissues. An epicardial layer (*Tbx18*) is usually observed immediately exterior to the mesenchymal tissue area and persists for greater time periods in multilineage organoids (still observable 2 months in culture), as opposed to conventional organoids where this layer diminishes by day 40. Smooth muscle tissue exhibiting slow, peristaltic contractions can be observed between the myocardium and endoderm as well [100,238,239].

Multilineage organoids exhibit greater tissue growth compared to organoids grown in a conventional medium, evidenced by an increase in the observed surface area by ~4000 μm^2^ (day 30). Furthermore, whilst organoids in conventional growth remain stagnant at ~2000 μm^2^, multilineage organoids continue to grow reaching a surface area of ~42,000 μm^2^ by day 100. This enhanced organoid growth can be attributed to cardiomyocyte proliferation evident by Ki67 staining, a marker of actively dividing cardiomyocytes. Not only does the presence of co-developing endoderm facilitate organoid growth and viability along with expansion and compartmentalization of cardiac tissue, but it also contributes to increased cellular heterogeneity and cardiac maturation. However, this increased cellular heterogeneity observed in multilineage organoids can be attributed in part to the presence of the various endoderm and primitive gut subtypes. A greater proportion of atrial/nodal cardiomyocytes is observed in multilineage organoids as well, a 16% increase over the percentage of atrial/nodal cardiomyocytes observed in conventional organoids which is timed with the appearance of endodermal mid-hindgut groups, thus highlighting their contribution to the specification of cardiac cell groups [100]. Cardiac maturation, facilitated by AA and calcium in the medium used, is evident by increases in cardiomyocyte perimeter and surface area as well as the identification of more elongated morphologies with increased sarcomere alignment and definition [100]. Furthermore, though many cardiac organoids contain endodermal lineages [8,101], long-term retainment of an epicardial layer along with greater specification towards atrial/nodal cardiomyocyte lineages has only been observed in this multilineage model [100].

Cardiac SCME models have also been created, allowing for the recapitulation of cardiac morphogenetic stages within the context of early embryonic developmental conditions [101]. They can be derived from starter PSC aggregations via an appropriate gastruloid medium [240] and additional cardiogenic linage direction with factors such as basic Fibroblast growth factor (bFGF), Vascular endothelial growth factor 165 (VEGF) and AA. Cardiac gastruloids exhibit characteristics consistent with the gastruloid stage of embryonic development, including anteroposterior patterning and axial elongation. Cardiac progenitor cells expressing *Mesp1* are usually identified at the anterior pole of the structure characterized by low levels of Wnt signaling, with FHF and SHF groups also specified in this model. FHF specification is marked by an abrupt increase (~4%) in cells characterized by *Hcn4* expression [241] while SHF specification is also similarly marked by an abrupt increase in *T-box transcription factor TBX1* (*Tbx1*) (~9%) expression [242], with both spikes in marker expression observed at 168 h in culture. Appearance of FHF and SHF populations further coincides with two peaks of *Mesp1+* expression occurring at 96 and 120 h in culture, while each peak is associated with a different trajectory of cellular movement, a phenomenon reflecting the migratory movement of FHF and SHF progenitors observed during development [101]. Morphologically, groups corresponding to cardiac populations transition from a spherical to a crescent and finally, concave shape while they also exhibit appropriate functionality evident by identification of spontaneous rhythmic Ca^2+^ transients. The cardiac groups in this model are adjacent to an anteriorly located epithelial endodermal group. An endocardial layer can be found in between the two, recapitulating the positioning and tissue interactions observed during in vivo embryogenesis. Cardiac gastruloids produced with this protocol generally contain various cell populations, including endothelial groups coalescing into vascular-like networks, additional mesoderm derivatives such as somitic formations, ectoderm derivatives as well as endoderm derivatives such as primitive gut formations [101].

Elongating multi-lineage organized gastruloids (EMLO) with cardiogenesis (EMLOC) are gastruloid models created to recapitulate co-development of cardiogenic and neurogenic lineages. Such models demonstrate not only cardiogenesis in the context of early embryonic development but interaction with a co-developing nervous system as well [102]. The constructs are generated from starter PSC populations via exposure to gastruloid medium (N2B27) [240] supplemented with cardiogenic factors (VEGF, AA, Fibroblast growth factor 2-FGF2) [101,102]. They assume an elongated shape with cardiac crescent formations eventually coalescing into a single heart tube with chambers. As expected, a higher percentage of EMLOCs exhibit cardiac crescent formations (~73%) compared to EMLOs (~2.7%), attributed to the use of cardiogenic factors in the former. These cardiogenic areas further increase with additional time in culture, which is evidenced by an increase in cTnT staining areas. Both FHF and SHF progenitor lineages are observed, although in day 16 EMLOCs, most cardiomyocytes are of the ventricular phenotype. The cardiomyocytes in the model further exhibit morphology associated with both proliferation/morphogenesis (round) as well as differentiation (mosaic). In particular, the mosaic cardiomyocyte population (66%) is larger by ~32% compared to actively proliferating round cardiomyocytes (34%), highlighting the simultaneously occurring processes of proliferation and differentiation. Cardiac chambers with spontaneous contractile function are also identified, evidenced by the identification of Ca^2+^ transients (day 7). As with other similar gastruloid models [101], cardiac regions are found anteriorly comprising endocardium, myocardium and epicardium, with cardiac jelly dividing endocardium and myocardium. Cardiac jelly degrades with time in the culture allowing for the generation of cardiac chambers [102].

Anterior endoderm regions can be seen adjacent to the anteriorly located cardiac regions. Co-developing neural regions can also be identified posterior to the cardiogenic and endoderm regions emerging on day 7, though spontaneous cardiac contractility is already present. The number of neuronal cells (*class III beta-tubulin-Tuj1*) and proportion of gastruloids with cardiac/neural integration from days 7 to 25 increases by 211 and 52.5, respectively, highlighting the ability of neuronal cells in this culture to invade and integrate within the developing cardiac regions. These neurons coalesce into ganglionic plexus formations from day 16 onwards, with contact between developing cardiomyocytes and neurons also visible [102]. Degradation of extracellular material within the cardiac jelly [243] allows invasion by inbound neuronal axons from nearby neurogenic regions. Although protocols comprising the separate derivation of cardiac and neuronal populations followed by co-culturing also exist [244,245,246], gastruloids combining cardiac and neural lineages allow for modeling of the interactions occurring between the two, as these would progress under developmental conditions [102].

Many of the previously described models can recapitulate development of vascular networks [8,9,89,97,99,100,101,102,196], although in some models vascularization can be specifically induced by appropriate protocols to better evaluate their effect on organoid structure, function and maturation parameters [53,103]. To this end, PSC-cardiomyocytes (comprising 80% of the total cell population in the final constructs), PSC-fibroblasts, PSC-epicardial and PSC-vascular cells can be combined in ratios corresponding to fetal heart tissue. The Vascularized cardiac organoids (hVCO) generated in this manner, though characterized by reduced surface area compared to human Cardiac organoids (hCO) owing to the decreased proportion of cardiomyocytes used, exhibit an increase in normalized beating rate (~20%) and force leading to overall enhanced contractile function. Furthermore, proteins related to angiogenesis, ECM organization, muscle structure and fibrillar function are upregulated, highlighting structural maturation [53]. Factors secreted by endothelial cells, including LAMA5 encoded by *laminin-α5*, facilitate this functional maturation due to the upregulation of mature *Troponin I* isoforms. In fact, *laminin-α5* expression in hVCOs is increased by 1.3-fold, a 30% increase compared to hCOs, while inducible KO of the *laminin-α5* gene leads to a 20% reduction in active force, highlighting its contribution to the increased force of contraction observed in hVCOs. This is further corroborated by experiments in murine models where *laminin-α5* mutations are associated with reduced muscle volume (82%), muscle area (96%) as well as myocardial wall thickness. Other factors with similar effects on vascularization such as platelet-derived growth factor (PDGF) similarly produce a 1.5-fold (PDGF-AB) and 1.9-fold (PDGF-BB) increase in force of contraction when added to hCOs. The favorable effect in this case is due to enhanced survival of stromal cells, production of ECM and pericyte recruitment. These in turn facilitate vessel formation and thus, enhance contractile function [53].

In similar studies, differentiated PSC-cardiomyocytes (CHIR99021, Wnt-C59) can be combined with PSC-vascular spheres (CHIR99021, VEGF) generating layered spherical vaschamcardioids (vcCO) [103]. While cardiomyocytes initially surround the more centrally located vascular spheres, endothelial cells from the center gradually move outwards (day 25), induced by a VEGF gradient, eventually leading to the formation of a central chamber found in ~80% of all organoids. The inner chamber lining is formed by a layer of cardiomyocytes intermixed with endothelial cells. Cardiomyocytes are the most abundant cellular population in vcCOs, comprising 71.76% of the total cell population (fetal heart cellular ratio) on day 25, followed by fibroblasts (17.97%), cardiac progenitors (4.95%) and endothelial cells (2.19%), although other non-cardiac cell types such as neurons and mesenchymal stem cells (MSC) are present as well. These cardiomyocytes are characterized by spontaneous beating in more than 90% of the vcCOs generated with this protocol. Evaluation of cellular interactions based on single-cell gene analysis reveals cardiomyocyte-endothelial interactions as well as cardiomyocyte–fibroblast interactions, mostly related to angiogenesis and the ECM. In particular, cardiomyocytes interact with endothelial cells via the Phosphoinositide 3-kinase (PI3K)/Protein kinase B (PKB or AKT) [247] and Ras signaling pathways as well as ECM-receptor signaling. The PI3K-AKT pathway favors cardiomyocyte growth and survival, while also facilitating endothelial cell migration and assembly of capillary networks. These characteristics, along with presence of cardiac fibroblasts contribute to cardiac tissue maturation [103,247,248].

Cardiac organoids can also be subjected to protocols aimed at deriving more mature structures [249], leading to production of cardiac organoids (CO) resembling fetal heart tissues and heart organoids (HO) resembling adult heart tissue ratios from starter EB aggregations [38]. While COs are produced via sequential induction (via GSK3 inhibition) and inhibition of the Wnt/β-catenin pathway, HO derivation involves administration of BMP, VEGF and TGFβ/Smad inhibitors allowing for specification of multiple lineages (cardiomyocytes, endothelial cells, fibroblasts). The organoids produced are generally spherical, with COs composed of 90% and HOs composed of 58% cardiomyocytes. While HO contain a lower percentage of cardiomyocytes, as expected of adult heart tissues, they contain increased numbers of cardiac fibroblasts (27%) and endothelial cells (15%) reflecting a more mature tissue composition. HOs also exhibit a higher beating efficiency compared to COs, which, although ranging from only 34.5% on day 8, it increases to 100% after 30 days in culture. COs, on the other hand, while exhibiting a higher initial efficiency at 68.1% (day 8), increase only to ~92.9% on day 30 [38] (Figure 2b).

## 5. Cardiac Models of Disease

Many of the constructs described in this text recapitulate aspects of embryonic and fetal cardiac development; as such, diseases and injuries relevant to embryonic and fetal systems shall be mostly analyzed. In some of these organoids the induced self-organization can be stimulated to progress further towards tissue compositions observed in the adult heart. This in turn allows for comparison of the responses to tissue injury between fetal- and adult-like heart tissues [38]. Cardiac morphogenesis is a process dependent on a complex interplay of genetic networks, many of which are active during early cardiac development. Some of these early expressed genes encode for cardiac TFs, in turn regulating the expression of other cardiogenic and vasculogenic factors and contributing to early developmental programs. Disruption in the expression of many of these genes can thus disrupt the normal progression of early development, leading to cardiac defects or complete arrest of cardiac morphogenesis [251].

*Tbx5* is a member of the T-box transcription gene family and it is involved in cardiac [252] and forelimb developmental programs [253,254]. Though TBX5 expression is initially widespread within the cardiac crescents, it eventually becomes more confined with higher expression identified in the posterior heart tube (sinus venosus, atria) [255]. It contributes to the development of both FHF and SHF populations [256], cardiac septation [257] and cardiac maturation. The protein TBX5 also interacts with the protein Nkx2.5, both acting synergistically to upregulate various downstream gene targets such as *Natriuretic peptide A (Nppa).* In addition, TBX5 forms transcriptional regulatory complexes with various other TFs, inducing the transcription of many different cardiac lineage genes [258]. *Tbx5* knockout (KO) in heart organoids affects mostly FHF lineages, with defects ranging from delayed appearance of the various morphogenetic stages (cardiac crescents, linear heart tube) to total failure of heart organoid formation. Chamber formation is affected [88] or absent as well [89]. Upregulation of aSHF gene expression also occurs along with downregulation of pSHF expression, reduction in LV organoid size and overall disruption in electrophysiological and contractile function [88]. *T-box transcription factor TBX1 (Tbx1*) is another gene member of the T-box transcription factor gene family involved in cardiac morphogenesis. In particular, it contributes to anterior heart tube elongation [28] and regulation of cardiac neural crest cell (cNCC) migration during OFT and aortic arch development [259,260,261]. *Tbx1* KO mainly disrupts the development of SHF lineages in associated precardiac organoids [90,262].

*Isl1* gene expression can be identified as early as E7, at least in embryonic murine models, during the cardiac crescent stage of cardiac morphogenesis [263]. *Isl1* encodes for ISL1, a LIM-homeodomain TF which can bind to and regulate the expression of various other TFs and epigenetic modifiers, participate in signaling networks involving BMP4 [264] and FGF10 [265], regulate FHF/SHF development [90,101,266,267] as well as expression of structural cardiomyocyte genes [268]. In addition, *Isl1* is often used as a marker for SHF groups [90]. As expected, *Isl1* KO results in the delay of cardiac morphogenesis with disruption in chamber formation and myocardial architecture [89]. In multichamber cardioids, *Isl1* KO causes a reduction in organoid size, more pronounced in OFT and atrial constructs. It also disrupts chamber formation and affects functional parameters such as organoid contractility [88].

ISL1 can also form DNA-binding complexes with Nkx2.5, another early cardiac TF implicated in cardiac morphogenesis, including FHF/SHF development [101,269]. Nkx2.5 is frequently used as a marker for cardiac progenitor populations [270], contributes to OFT development [271] and along with *Hand1*, cardiac chamber formation [95,96,272,273]. In addition, Nkx2.5 potentiates Wnt signaling by modulating R-spondin3 expression, a Wnt signaling agonist [274] and contributes to the specification of the cardiac conduction system [273]. While constitutive absence of *Nkx2.5* expression is lethal, conditional absence (only in specific tissues or under specific conditions) can lead to more specific effects. For example, conditional absence of *Nkx2.5* in ventricular cells leads to disruption in the specification of the cardiac conduction system as well as ventricular trabeculation [29]. *Nkx2.5* KO in cardiac organoids also generally leads to disruption in myocardial compaction, size and tissue architecture. Smooth muscle proliferation is increased as well, as the repressive signals on differentiation of smooth muscle are removed [8,275].

*Forkhead box protein F1 (FoxF1)* is a member of the Forkhead transcription factor family and is involved in the regulation of vasculogenic/angiogenic programs [276], pSHF lineage development and cardiac septation [88]. *FoxF1* KO thus results in early embryonic lethality, disruption in pSHF lineage specification as well as organoid size and contractile activity [88] (Table 2).

Environmental factors, including exogenously administered pharmaceutical substances, can also affect cardiac development. Various compounds have been tested for their effects on cardiac development including antibiotics (amoxicillin, rifampicin, doxycycline), antihistamines (doxylamine succinate) [92], lithium carbonate and phenytoin [277]. Some of these agents, such as lithium carbonate, are involved in the inhibition of phosphatidylinositol recycling [278] as well as the stimulation of Wnt/β-catenin signaling [279,280,281], possibly explaining their effects on organoid area and growth [92]. Others, including various antibiotics, can affect cellular proliferation [282] and thus inhibit cardiac differentiation (doxycycline) [92,283] or lead to developmental arrest at high enough concentrations (rifampicin) [92].

The effect of compounds historically associated with congenital defects such as thalidomide [284] has also been examined along with chemotherapeutic compounds (doxorubicin) [92,103]. Thalidomide is teratogenic for human embryo development affecting, among others, DNA-binding of TBX5, TBX5-Hand2 binding [39] and early mesendoderm specification [285]. As a result, TBX5 gene targets such as *Nppa*, *Vegf* are downregulated [286], eventually leading to various effects on cardiac development [39]. Doxorubicin is a chemotherapeutic anthracycline compound [287] used in treatment regimens for solid tumors, leukemia and lymphoma [288]. It is frequently associated with cardiotoxicity which can in turn lead to chronic cardiomyopathy and heart failure [288,289]. In organoid models it mainly affects contractile and electrophysiological activity, with effects on apoptosis also observed [103].

Ondansetron is a 5-HT3 receptor antagonist used to treat severe nausea and vomiting during pregnancy (NVP) [290]. While some epidemiological studies associate ondansetron with congenital defects including ventricular septal defects (VSD) [291], others produce no significant association [99,290]. In cardiac organoid models, ondansetron mainly leads to disruption of contractile and electrophysiological activity as well as myocardial morphology, although no effects on apoptosis are observed. The observed disruption of ventricular chamber morphology could allude to the VSDs often associated with clinical ondansetron use in some studies [99,292].

Retinoid compounds are used for the treatment of various conditions including psoriasis and other skin problems as well as leukemia [88]. RA (all-trans RA) is involved in the anteroposterior patterning of cardiac mesoderm and as such, is expected to affect cardiac morphogenesis upon perturbation of its concentration gradient during development [195,197]. Various retinoid compounds have been tested in relevant models including all-trans retinol and acitretin. Retinoids generally produce severe effects even with minimal doses, ranging from effects on tissue patterning and cell linage specification to disruption in cardiac morphogenesis. Effects on electrophysiological activity and organoid growth are also observed [92]. In multi-chamber organoid models, in particular, retinoids can lead to varying effects depending on phenotype with some chambers (OFT) affected more than others [88].

Organoids can thus be useful as platforms for developmental toxicity testing, though these effects are tested on constructs resembling early embryonic stages. However, use of maturation protocols [38] to reach states of cellular assembly seen later during development can aid in distinguishing between different effects in tissues of different maturity. This can in turn allow for comparison between early and late developmental effects of the same compound, while high volume production of cardiac organoids can also be useful for evaluating these effects amongst a high number of replicates [6,88,92,99,103]. Though many of these substances induce similar effects in both organoid models and zebrafish whole embryo cultures (zWEC), others, including rifampicin, doxycycline and thalidomide, produce milder effects in organoids. Some authors attribute this phenomenon to interspecies differences, variations in drug administration and different range of effective treatment concentrations for each system [92,293]. The extent to which this can be attributed to species differences or other additional factors, however, must be fully evaluated with additional studies in order to increase available data on the developmental toxicity of various compounds [92] (Table 3).

Cardiac organoids can also be used to model pathophysiological tissue processes associated with complex disease conditions including myocardial infarction (MI) [38], diabetes mellitus (DM) [9], cardiac hypertrophy [98], post-injurious fibrosis [103,294] and inflammatory tissue injury [53]. In some systems ischemic injury can be recapitulated via a cryoinjury model used to simulate localized cardiomyocyte tissue loss with preservation of viability in the surrounding cells [95,295,296]. Local tissue compaction and necrosis usually ensue while accumulation of extracellular material occurs in the affected areas, composed mainly of fibronectin and fibroblasts secreting COL1A1. Fibroblasts in these models are usually derived from epicardial groups and accumulate in injured organoids [95].

In similar cryoinjury models, cardiomyocyte loss can also be observed, corroborated with assays evaluating for the release of LDH and cardiac troponin I (cTnI) revealing a 3-fold increase in both, compared to control conditions. As these models are mainly recapitulating fetal heart conditions, the post-injury fibrosis and hypertrophy are less than what would be expected in adult cardiac tissues. Thus, no accumulation of fibronectin occurs post-injury along with no change in total cardiomyocyte area. In addition, genes associated with cardiac hypertrophy such as *Nppa* and *Acta1* show no significant upregulation as well. Cardiomyocyte proliferation is another characteristic of immature tissues, although no significant change before and after injury is observed in this model, although the higher baseline levels of cardiomyocyte proliferation usually present in such tissues is evident. After injury, local cardiomyocytes resume normal function within 2 weeks, another characteristic attributed to local cardiomyocyte regeneration [296].

Vascularized organoids can also be subjected to cryoinjury in order to simulate myocardial ischemia, MI and the post-injurious fibrotic response. After injury is induced, electrophysiological function is affected while contractile function becomes asynchronous amongst different cardiomyocyte groups. Markers of cardiomyocyte become elevated (cTnT) while a fibrotic response ensues affecting ~40% of total organoid area with elevation in relevant markers such as Vimentin (*Vim*) and *alpha Smooth muscle actin (α-SMA).* While administration of captopril, an angiotensin-converting enzyme inhibitor (ACEi), restores relative mRNA levels of endothelial, cardiomyocyte and fibrotic marker genes to those observed pre-injury, it does completely reverse tissue fibrosis. However, it restores electrophysiological function, including Ca^2+^ transient amplitude and contraction synchronization. Organoid vascularization is useful for the more accurate recapitulation of these effects, as organoids without vascularization exhibit a lower degree of fibrosis while the favorable properties of captopril on the mitigation of this response are not seen as well [103,294].

MI-associated ischemia-reperfusion (IR) injury, along with the resulting myocardial fibrosis, can also be recapitulated in CO models (fetal-like tissue) and HO models (adult-like tissue) [38]. In this case, the inciting injury is induced via administration of cobalt-chloride (CoCl_2_) and glucose depletion. IR injury can be then recreated by subjecting organoids to high glucose and Ca^2+^ concentrations, as relevant studies relate magnitude of IR to the glucose-induced sensitization [297] and intracellular/intra-mitochondrial Ca^2+^ overload [298] occurring during this time. As a result, apoptosis can be observed with reduction in cardiomyocytes and associated cTnT staining, more pronounced in HO constructs. This is further validated by a greater decrease in intracellular markers of injury such as intracellular cTnT, cTnI and greater increase in secreted markers such as cTnI, myoglobin (MB) and creatinine kinase (CKM). In COs, on the other hand, the increase is either not significant (as is the case with cTnI and CKM) or not as high. The sarcomere disintegration, inflammatory response triggered by the release of nuclear factor of kappa light polypeptide gene enhancer in B-cells (NF-κB) and post-infarction remodeling is also more enhanced in HOs compared to COs. While fibrosis post-MI can be recapitulated in both organoid models with culturing in the presence of TGFβ, this phenomenon is more pronounced in HO organoids as well [38].

hHOs can also be used to model the effects of maternal diabetes mellitus (Gestational diabetes mellitus—GDM) on embryonic/fetal development [9]. GDM affects fetal metabolism leading to abnormalities in glucose and lipid metabolic pathways, as large amounts of glucose are often required to maintain normal metabolic activity. Fetal hypoglycemia eventually ensues, disrupting brain function due to the inadequate glucose levels, leading to increased tissue growth [299] and causing cardiovascular disruption. Cardiac events in GDM can be recapitulated in vitro via glucose and insulin modulation. Morphologically, though hHOs grown under normal conditions exhibit elongation/patterning, hHOs recapitulating GDM conditions remain spherical and are larger overall. Additional characteristics include electrophysiological irregularities (arrhythmia), increased rate of glycolysis with reduced mitochondrial numbers, reduced oxygen consumption and increased numbers of lipid droplets (lipid dysregulation). Mean numbers of cardiomyocytes are affected as well, with mean ventricular cardiomyocyte ratios reduced by ~10% and mean atrial cardiomyocyte ratios increased by ~25%, reflecting the cardiac structural disruption in fetal tissues often associated with GDM. Epicardial groups are also abnormally localized in organoids recapitulating disease conditions, as they are surrounded by myocardial tissue, compared to their usual location adjacent to myocardial groups [9].

CCOs can be modified to recapitulate aspects of cardiac disease through administration of endothelin-1 [98], a vasoconstrictor and known inducer of cardiac hypertrophy. Cardiac hypertrophy is induced via MAPK signaling pathways [300], resulting in pathologic hypertrophic remodeling [301] associated with upregulation of fetal genes (*Nppa*, *Natriuretic peptide B-Nppb*) and downregulation of adult protein isoforms (α-*Myosin heavy chain 6-αMhc6)* relevant to fetal protein isoforms (β-*Myosin heavy chain- βMhc7*) [300]. Endothelin-1 administration leads to alterations in myocardial tissue structure, including aspects of actin–myosin interactions, leading to myofibrillar disarray. The myocardial hypertrophy associated with endothelin-1 is relatively minor, although with high concentrations the increase in wall thickness is sustained weeks after treatment. With lower treatment doses, myocardial thickness generally reverts to baseline conditions after 1 week. Contractile abnormalities, namely contraction frequency and variability are also seen, along with electrophysiological disruption. Fractional shortening, reflecting left ventricular function, also decreases with increased endothelin concentrations, highlighting the detrimental effects of endothelin on ventricular structure and function [98].

Organoids modified with vascularization protocols (hVCO) can also be useful for the modeling of inflammatory disease conditions, including recapitulation of the effects of cytokine storm (CS) on cardiac function [53]. Recreation of a cytokine storm (CS) in vitro entails use of inflammatory factors such as interferon γ, IFN-γ, poly(I:C) and interleukin-1β (IL-1β). The magnitude of the observed inflammation and resulting diastolic dysfunction is proportional to the level of endothelial networks observed in the organoids tested. Evaluation of the effects of endothelin-1 on hVCO function similarly reveals an increased time to relaxation along with an increase in contractile force and rate of contraction. Endothelin-1 is often increased with various inflammatory conditions [302,303], including COVID-19 infections [53]. It binds to endothelin receptors, such as Endothelin receptor A commonly expressed in pericytes and vascular smooth muscle cells (VSMC) of the heart, increasing smooth muscle relaxation time and bringing about chronic diastolic dysfunction. In the organoid models, administration of endothelin-1 antagonists (bosentan, sitaxsentan) mitigates these effects, highlighting the role of this substance as a mediator of the pathologic effects due to inflammation on contractile function [53,304] (Table 4).

Most of the diseases or tissue insults modeled with these organoid systems are primarily diseases affecting embryonic/fetal systems, owing to the embryonic/fetal composition of the organoids involved. For example, genetic knockouts have been applied in many of the cardiac models described in this text in order to evaluate the role of the genes affected. Since many of these encode for proteins that function as TFs, their downregulation or absence can be associated with many deleterious effects on cardiac development, which can range from early embryonic lethality or complete developmental arrest to widespread effects on cardiac gene transcription. Furthermore, while these studies are useful at organoid level, the widespread nature of these genetic knockouts effect might not be as easy or ethical to replicate in living organisms [8,88,89,90]. Perhaps additional organoid studies on the effects of genetic knockout in genes with more specific actions could be useful. Furthermore, as congenital heart defects are often associated with multiple mutations, standalone study of each one of these in organoid systems might further help elucidate their contribution to pathologic phenotypes [305].

## 6. Conclusions

While EB is a term used to refer to PSC conglomerations without specific organizational characteristics, various tissue models and structures can be derived from these, including SCMEs and organoids resembling embryonic/fetal and adult tissue. Organoids, in particular, though generally recapitulating adult-like structures, represent embryonic/fetal tissue states when it comes to cardiac systems. Various cardiac organoid models have been produced in the last years, most of which are derived from starting PSC populations. As all these constructs are mostly recreations of embryonic/fetal-like structures, modeling the effects of disease or various pharmaceuticals generates effects usually observed in immature tissues, making such models better suited to study genetic and congenital disease as well as disease associated with developmental toxicity. However, it is important to further improve upon and standardize organoid SCME protocols, allowing for easier comparison between different studies to more effectively recreate cardiac tissue and disease in vitro.

## Figures and Tables

**Table 2:** Summary of the key characteristics of the various cardiac organoids systems used to recapitulate genetic and developmental disease. Ref, Reference; Tbx5, T-box transcription factor TBX5; Tbx1, T-box transcription factor TBX1; Tbx2, T-box transcription factor TBX2; KO, knockout; EB, Embryoid Body; hPSC, human Pluripotent stem cells; hiPSC, human induced Pluripotent stem cells; CCL, Cardiac crescent-like; HT, Heart tube; LHT, Looping heart tube; CF, Chamber formation; WT, Wild type; LN, Laminin; ET, Entactin; WT, Wild type; cTnT, cardiac Troponin T; FGF2, Fibroblast growth factor 2; Fgf10, Fibroblast growth factor 10; CHIR99021, Chiron; FGF4, Fibroblast growth factor 4; BMP4, Bone morphogenetic protein 4; LIF, Leukemia inhibitory factor; BIO, 6-bromoindirubin-3-oxim; IWP2, Inhibitor Wnt production-2; Myh11, Myosin heavy chain 11; Nr4a3, Nuclear receptor subfamily 4 group A member 3; Nppa, Natriuretic peptide A; WNTi, Wingless-related integration site (Wnt) inhibitor; RA, Retinoic acid; Irx4, Iroquois-class homeodomain protein irx-4-A; Nr2f2, Nuclear receptor subfamily 2 group F member 2; TGFβi, Transforming growth factor beta inhibitor; Myl7, Myosin light chain 7; Pitx2, Pituitary homeobox 2; Eno1, Enolase 1; Wnt5A, Protein Wnt-5A; Nkx2.5, Homeobox protein Nkx-2.5; Isl1, Insulin gene enhancer protein Islet-1; SHF, Second heart field; Hey1, Hairy/enhancer-of-split related with YRPW motif protein 1; aSHF, anterior Second heart field; pSHF, posterior Second Heart field; FHF, First heart field; WT, Wild type; cTnT, cardiac Troponin T; HFO, Heart-forming organoid; OFT organoid, Outflow tract organoid; HoxB1, Homeobox protein Hox-B1; RV, Right ventricular organoid; LV, Left ventricular organoid; AVC, Atrioventricular organoid; Actc1, Actin, alpha cardiac muscle 1; CXCR4, C-X-C Chemokine receptor type 4; Tnnt2, Troponin T; Mef2c, Myocyte-specific enhancer factor 2C; Myocd, Myocardin; FoxF1, Forkhead box protein F1; FoxC2, Forkhead box protein C2; Hand1, Heart- and neural crest derivatives-expressed protein 1; Hand2, Heart- and neural crest derivatives-expressed protein 1; bmp, beats per minute; Rspo3, R-spondin3; Gata4, Transcription factor GATA-4.

**Gene**	**Effects**	**Organoid**	**Ref.**
*Tbx5* KO	Delayed cardiac morphogenesis (25% present at the CCL stage on day 3 versus ~30% at the CCL stage for WT), delay/absence of chamber formation (no chambers for 2 of the 3 KO lines used, 8.3% at the CF stage in only one line on day 9 versus ~40% at the CF stage for WT), *Tbx5* expression decreased, *Tbx5/Nkx2.5* localization disrupted, myofibrillar architecture disruption (weaker cTnT immunofluorescence for KO line versus WT).	Starter material: EB (ESCs) Factors: LN/ET, FGF4, BMP4, BIO, LIF	[89]
*Tbx5* KO	Atrial: aSHF marker upregulation, pSHF marker (*HoxB1*) downregulation, cardiomyocyte differentiation efficiency reduced with *Tnnt2*, *Nppa* downregulation, ventricular chamber marker (*Irx4*) downregulation, loss of spontaneous contractile activity.	Starter material: hPSCs Factors: Mesoderm (FGF2, BMP4, Activin A, CHIR99021), FHF-derived (LV) (BMP4, FGF2, Insulin, WNTi, RA), aSHF-derived (RV, OFT) (WNTi, TGFβi, RA) pSHF-derived (Atrial, AVC) (WNTi, TGFβi, RA)	[88]
	AVC: cardioid area reduced to almost 0 (day 9.5), aSHF marker upregulation, pSHF marker (*HoxB1*) downregulation, cardiomyocyte differentiation fails on day 9.5, ventricular chamber marker (*Irx4*) downregulation, loss of spontaneous contractile activity.		
	LV: cardioid area reduced by ~4,000,000 μm^2^ (day 9.5), dysregulated gene expression with upregulation of *Hand2*, *Fgf10*, *Tbx2*, *Wnt5a*, downregulation of *Nkx2.5*, *Gata4*, cardiomyocyte differentiation efficiency reduced (*Tnnt2*, *Nppa* downregulation), loss of spontaneous contractile activity, ventricular chamber marker (*Irx4*) downregulation.		
	RV: cardiomyocyte differentiation efficiency reduced with *Tnnt2*, *Nppa* downregulation, upregulation of *FoxC2*, *Tbx2*, *Wnt5a*, ventricular chamber marker (*Irx4*) downregulation, loss of spontaneous contractile activity.		
*Tbx5* KO	Development of FHF (CXCR4-) lineages (LV) affected (0.003 decrease in normalized expression of *actc1*, no effect on *actc1* expression in CXCR4+ lineages).	Starter material: hiPSCs Factors: BMP4, CHIR99021	[90]
*Tbx1* KO	Development of SHF (CXCR4+) (aSHF: RV, OFT; pSHF: atria, AVC) lineages affected (~0.5 and ~0.7 decrease in relative proliferation of CXCR+ cells after 24 and 48 h, respectively, no effect on relative proliferation of CXCR4- lineages).		
*Isl1* KO	Delayed cardiac morphogenesis (16.7% present at the CCL stage on day 3 versus ~30% at the CCL stage for WT, no HT stage for 1 of 3 KO lines used), delay/absence of chamber formation (no chambers for 2 of 3 KO lines used, 12% at the CF stage in only one line on day 9 versus ~40% at the CF stage for WT), no clear separation of *Tbx5/Nkx2.5* immunofluorescence, myofibrillar architecture disruption (weak cTnT immunofluorescence for KO line versus WT).	Starter material: EB (ESCs) Factors: LN/ET, FGF4, BMP4, BIO, LIF	[89]
*Isl1* KO	OFT: cardioid area reduced by ~900,000 μm^2^ (day 9.5), downregulation of *Hand2*, BMP4, upregulation of *Tbx5*, misregulation of *Nr2f2*, *Rspo3*, *Wnt5a*, *Myl7*, cardiomyocyte differentiation efficiency reduced (only some *Tnnt2* immunofluorescence present) with downregulation of *Mef2c*, *Myocd* (cardiac differentiation genes), global shift towards atrial phenotype (positive change in expression of *Nr2f2*, *Hey1*, negative change in expression of *Wnt5a*), severe impairment in chamber formation by day 5.5.	Starter material: hPSCs Factors: Mesoderm (FGF2, LY, BMP4, Activin A, CHIR99021), FHF-derived (LV) (BMP4, FGF2, Insulin, WNTi, RA), aSHF-derived (RV, OFT) (WNTi, TGFβi, RA) pSHF-derived (Atrial, AVC) (WNTi, TGFβi, RA)	[88]
	Atrial: cardioid area reduced by ~900,000 μm^2^ (day 9.5), downregulation of *HoxB1* (pSHF), misregulation of *Nr2f2*, *Rspo3*, *Wnt5a*, *Myl7*, cardiomyocyte differentiation efficiency reduced (some *Tnnt2* immunofluorescence present) with downregulation of *Mef2c*, *Myocd* (cardiac differentiation genes), reduced spontaneous contractile activity (bpm decrease by 10, day 9.5), severe impairment in chamber formation by day 5.5.		
	RV: cardioid area only slightly reduced (day 9.5), misregulation of *Nr2f2*, *Rspo3*, *Wnt5a*, *Myl7*, cardiomyocyte differentiation efficiency reduced (almost no *Tnnt2* immunofluorescence) with downregulation of *Mef2c*, *Myocd* (cardiac differentiation genes), effects on chamber formation (day 5.5) less severe.		
	LV: cardioid area reduced by ~2,000,000 μm^2^ (day 9.5), misregulation of *Nr2f2*, *Rspo3*, *Wnt5a*, *Myl7*, cardiomyocyte differentiation efficiency not as affected (*Tnnt2* immunofluorescence present) with *Mef2c*, *Myocd* (cardiac differentiation genes) upregulated, effects on chamber formation less severe.		
*Nkx2.5* KO	Total organoid area increased by ~1 mm^2^ (day 10), reduction in myocardial layer compaction by 20% compared to control (70%) (day 10) though with maximum compactness similar to control reached by day 13, disruption of intercellular cardiomyocyte adhesions, cardiomyocyte hypertrophy (increased in cardiomyocyte area by ~0.1 mm^2^), 41.7-fold and 25.9-fold downregulation of NKX2.5 gene targets (*Nppa* and *Irx4,* respectively), smooth muscle proliferation (15-fold increase in *Myh11* upregulation, 11-fold increase in *Nr4a3* upregulation).	Starter material: hPSC Factors: CHIR99021, IWP2	[8]
*FoxF1* KO	LV: cardioid area reduced by ~1,600,000 μm^2^ (day 9.5), downregulation of *Eno1* (involved in cardiac contraction), *HoxB1*, *Tbx5* (pSHF) with complete absence of pSHF specification, *Isl1*, *Tbx1* (aSHF), upregulation of *Pitx2*, *Tbx1*, reduced spontaneous contractile activity (bpm decreased by 10, day 6.5).	Starter material: hPSCs Factors: Mesoderm (FGF2, LY, BMP4, Activin A, CHIR99021), FHF-derived (LV) (BMP4, FGF2, Insulin, WNTi, RA), aSHF-derived (RV, OFT) (WNTi, TGFβi, RA) pSHF-derived (Atrial, AVC) (WNTi, TGFβi, RA)	[88]
	AVC: cardioid area reduced by ~500,000 μm^2^ (day 9.5), cessation of spontaneous contractile activity (day 6.5), no chamber formation.		
	Atrial: cardioid area reduction not significant (day 9.5), downregulation of *HoxB1* (pSHF) with complete absence of pSHF specification, *Isl1*, *Tbx1* (aSHF), shift towards ventricular phenotype with extensive chamber formation, reduced spontaneous contractile activity (bpm decrease by ~6, day 6.5), earlier chamber formation (day 3.5).		
	RV: cardioid area reduction not significant (day 9.5), downregulation of *Nppa*, *HoxB1*, *Tbx5* (pSHF) with complete absence of pSHF specification, upregulation of *Pitx2*, *Tbx1.*		
	OFT: cardioid area reduction not significant (day 9.5), downregulation of *Nppa.*		

**Table 3:** Summary of the key characteristics of the various cardiac organoids systems used to evaluate the effects of pharmaceutical compounds on development, as described in this manuscript. Ref, Reference; zWEC, zebrafish Whole embryo culture; AVC, Atrioventricular; OFT, Outflow tract; RV, Right ventricular; LV, Left ventricular; Tbx5, T-box transcription factor TBX5; Nppa, Natriuretic peptide A; Wnt, Wingless-related integration site; Wnt5A, Protein Wnt-5A; FWHM, Full-width at half-maximum; Irx1, Iroquois-class homeodomain protein irx-1-A; Irx4, Iroquois-class homeodomain protein irx-4-A; Nr2f2, Nuclear receptor subfamily 2 group F member 2; GFP, Green fluorescence protein; Tnni1, Troponin I 1; Myl7, Myosin light chain 7; Myl2, Myosin light chain 2; RA, Retinoic acid; LV, Left ventricular; 5-HT3, 5-Hydroxytryptamine 3 (serotonin AP, Action potential; VSD, Ventricular septal defect; AP, Action potential; TUNEL, Terminal deoxynucleotidyl transferase dUTP nick end labeling.

Compounds	Effects	Ref.
Doxylamine succinate	No effect in cardiac differentiation efficiency, some increase in cardiac tissue (increase in area ratio by ~0.1 with 1 μM), reduced cardiac tissue compaction, decrease in contraction velocity (by ~5 μm/s with 10 μM) and beating rate (by ~10 bpm with 10 μM), similar effects in zWECs	[92]
Amoxicillin	No effects on organoid function and structure, similar effects in zWECs	
Rifampicin	Arrest of organoid development with high concentrations (100 μM), milder effects in zWECs	
Doxycycline	Inhibits cardiac differentiation and cardiac organoid formation (area ratio, organoid height reduced to 0 with 10 and 100 μM, respectively), milder effects in zWECs	
Lithium carbonate	Stimulates the Wnt/β-catenin signaling pathway, affects organoid formation with reduction in area ratio (median reduced by 0.1 with 10 μM) and FWHM (median reduced by ~50 with 10 μM), no effects in contractile function, similar effects in zWECs	
Phenytoin	Reduction in overall organoid size with decreases in area ratio (median reduced by 0.1 with 10 μM), height (~100 μm with 10 μM) and FWHM (~100 μm with 10 μM), cessation of contractile activity with high doses (100 μM), no disruption in normal tissue architecture, similar effects in zWECs	
Tretinoin (All-trans RA) Isotretinoin	No effect/increase in organoid size but with failure of cardiac differentiation (area ratio 0 with 10μM tretinoin) (area ratio 0 with 1μM isotretinoin), cessation of contractile activity (10μM tretinoin) (1 μM, 10 μM isotretinoin), similar effects in zWECs	
Acitretin	Severe effects on lineage specification/tissue patterning/cardiac morphogenesis on atrial/AVC/OFT, effects on atrial size with 5 nM (day 4.5) and AVC size (50 nM) (day 4.5), severe effect on OFT size (cardioid area reduced by ~2,000,000 μm^2^) (50 nM) (day 9.5), severe effect on LV/RV size (cardioid area reduced by ~3,000,000 μm^2^) (50 nM) (day 9.5), disruption in chamber formation in atrial/AVC/OFT (*Tnni1-GFP* imaging) (day 9.5), downregulation of OFT-specific genes (*Wnt5a*), upregulation of ventricular genes (*Irx1*, *Irx4*) in OFTs, earlier cardiomyocyte differentiation in OFTs	[88]
All-trans retinol	Severe effects in tissue morphology in OFT with no effects on other organoids, downregulation of OFT-specific genes (*Wnt5a*), upregulation of ventricular genes (*Irx4*, *Irx1*) in OFTs, earlier cardiomyocyte differentiation in OFTs	
Thalidomide	Impairs early mesendoderm specification, reduction in cardiac differentiation efficiency (progressive reduction in differentiation efficiency, efficiency reduced to 10.83% with 100 μM compared to 63.52% for control), disorganized organoid morphology with reduced size parameters (greatest median height reduction by ~100 μm with 1 μM, greatest median FWHM reduction by ~100 μm with 100 μM), reduced cardiac differentiation (greatest reduction in median area ratio by ~0.2 with 100 μM), no effect on contraction velocity, heart rate variability (increase by ~5 bpm with 1 μM, followed by progressive decrease with increasing dose), milder effects in zWECs	[92]
	Severe phenotype in AVC (effects on AVC size with 0.1 μg/mL), intermediate phenotype in LV/RV (progressively decreasing size, greatest effect with 10 μg/mL), subtle phenotype in atrial/OFT (progressively decreasing size, greatest effect with 10 μg/mL), downregulation of *Nppa* (downstream of TBX5) in atrial/AVC/LV, *Nr2f2* upregulation in AVC (atrial marker), *Irx1* downregulation in RV (RV marker), *Irx4* downregulation in LV (LV marker)	[88]
Doxorubicin (anthracycline)	Cardiotoxicity (progressing to cardiomyopathy, congestive heart failure), effects on contractile activity (complete cessation of beating rate when 10 μM applied for 72 h, 50 μM applied for 48 h), decrease by 2 ΔF/F0 in peak Ca^2+^ amplitude and increase by 0.5 sec in time to peak (1 μM, 10 μM), decreasing cell viability with increasing dose, increasing cell apoptosis (~40% increase in TUNEL+ cells) with increasing dose	[103]
Ondansetron (5-HT3 receptor antagonist)	Electrophysiological abnormalities (progressive decrease in beating rate, decreased frequency/amplitude of AP with increasing concentrations due to Na+ channel blockade, QT prolongation), total area occupied by atrial cells unaffected (*Myl7*), total area occupied by ventricular cells (*Myl2*) reduced to 0.55-fold, 0.18-fold with 10 μM, 100 μM, respectively, (compared to control), structurally disorganized ventricular chambers (loss of ventricular wall definition, loose chamber separation seen with 100 μM), *Myl2* expression decreased to 0.40-fold with 100 μM (compared to control), no effect on apoptosis	[99]

**Table 4:** Summary of the key characteristics and results of the various cardiac organoids systems used to recapitulate tissue injury. Ref, Reference; MI, Myocardial infarction; ECM, Extracellular matrix; COL1A1, Collagen alpha-1(I) chain; hCO, human Cardiac organoids; GDM, Gestational Diabetes mellitus; CH, Cardiac hypertrophy; CHD, Congenital heart defect; CH, Cholesterol; TG, Triglycerides; LDL, Low-density lipoprotein; CCO, Chambered cardiac organoids; hVCO, human Vascularized Cardiac Organoids; CS, Cytokine storm; IFN-γ, Interferon-γ; poly(I:C), Polyinosinic:polycytidylic acid; IL-1β, Interleukin-1; VSMC, Vascular smooth muscle cells; α-SMA, alpha Smooth muscle actin; Vim, Vimentin; PECAM1, Platelet endothelial cell adhesion molecule 1; Cdh5, Cadherin 5; Tnnt2, Troponin T; Myh7, Myosin heavy chain 7; cTnT, cardiac Troponin T; NF-κB, nuclear factor of kappa light polypeptide gene enhancer in B-cells; HIF-1a, Hypoxia inducible factor 1 alpha; IR, Ischemia reperfusion; CO, Cardiac organoid; TGF-β1, Transforming growth factor beta 1; AMI, Acute myocardial infarction; cTnI, cardiac Troponin I; MB, Myoglobin; CKM, Creatine kinase M; ERK, Extracellular signal regulated kinase; JNK, c-Jun N-terminal kinases; SERCA, Sarcoplasmic/endoplasmic reticulum calcium ATPase; HO, Heart organoid.

Injury	Effects	Ref.
MI (cryoinjury model)	Local tissue compaction, necrosis and limited apoptosis (TUNEL+ staining cells), extracellular material accumulation composed of fibronectin and COL1A1-secreting fibroblasts, fibroblasts derived from epicardial lineages in tri-lineage models (cardiomyocytes, epicardial cells, cardiac fibroblasts), no COL1A1/fibroblasts and reduced fibronectin accumulation in single lineage models (cardiomyocytes)	[95]
MI (cryoinjury model)	Localized cardiomyocyte loss (~15% reduction in GFP-cardiomyocyte expression) associated with 3-fold increase in secreted LDH and cTnI compared to control, post-injury fibrosis (no increase in fibronectin accumulation, effects reduced compared to adult tissues), no significant post-injury hypertrophy (no change in cardiomyocyte area, no significant upregulation in *Nppa*, *Acta1* expression), quantification of cardiomyocyte proliferation (Ki67, pH3) shows variation between cell lines, higher baseline levels of cardiomyocyte proliferation overall (tissue immaturity) with return to baseline function after 2 weeks	[296]
MI, Fibrosis (cryoinjury model)	Localized cardiomyocyte loss associated increase in secreted cTnT (from ~25 pg/mL to 125 pg/mL), effects on electrophysiological activity with 40% decrease (decrease from ~5 to 3 ΔF/F0) in amplitude (ΔF/F0) and time to peak (s) increase to ~0.6 s (Ca^2+^ transients), asynchronous contractile function amongst cardiomyocyte groups, 40% increase in total fibrotic area, upregulation of fibrosis markers (*Vim*, *α-SMA*), downregulation of endothelial (*PECAM1*) and cardiomyocyte (*Myh7*, *Tnnt2*) markers, lower degree of fibrosis (~20% total fibrotic area) in non-vascularized organoids	[103]
MI, Fibrosis (cryoinjury model, captopril)	Captopril (ACEi) administration restores endothelial, cardiomyocyte and fibrotic marker expression to about pre-injury levels, reversal of fibrotic area not to pre-injury levels (although improved to ~20% of total organoid area), mitigation of electrophysiological disturbances (Ca^2+^ transient amplitude), synchronization in contractile function amongst cardiomyocyte groups, no favorable effects of captopril administration in non-vascularized organoid fibrosis (non-significant effect on % total fibrotic area)	
MI, IR, Fibrosis (CoCl_2_ and Glc depletion, high Glc and Ca^2+^, TGFβ)	Cardiomyocyte apoptosis (tissue response not as extensive as observed in mature organoids, non-significant increase in cTnI, CKM release and only 10-fold increase MB release from affected cells), less pronounced sarcomere disintegration, inflammatory response (NF-κB), post-infarction remodeling and post-injury fibrosis	[38]
MI, IR, Fibrosis (CoCl_2_ and Glc depletion, high Glc and Ca^2+^, TGFβ)	More accurate recreation of MI injury HO constructs, greater decrease in intracellular cTnT, cTnI, greater increase in cTnI (up to 15-fold in 72 h compared to controls), CKM (2-fold in 72 h compared to controls), MB (up to 20-fold in 72 h compared to control) release from affected cells, more pronounced sarcomere disintegration and inflammatory response (NF-κB), post-MI remodeling (upregulation of ERK, JNK, p38, SERCA, alterations in Ca^2+^ handling), more pronounced post-MI fibrosis with effects on contractile and electrophysiological activity	[38]
GDM (Glc, insulin modulation)	Absence of elongation/tissue patterning (4–8 days) with spherical shape and increase in overall size (organoid area at ~1,500,000 µm^2^ versus less than ~1,500,000 µm^2^ for controls) (4–8 days), electrophysiological irregularities (mean beating rate increased by 10 bpm), increased rate of glycolysis, reduced mitochondrial numbers (~0.25 mean mitochondria/μm^2^ versus mean number of 0.5 mitochondria/μm^2^ in controls), reduced O_2_ consumption, increased numbers of lipid droplets, disruption in cardiomyocyte populations (mean ventricular cardiomyocyte ratio decreased by ~10%, mean atrial cardiomyocyte ratio increased by ~25%), abnormally localized epicardium	[9]
CH (Endothelin-1)	Disruption in myocardial tissue architecture and actin–myosin interactions (skewed sarcomeric z-lines, myofibrillar disarray), myocardial hypertrophy (increase in thickness ~40%, sustained for 3 weeks with 100 ng/mL, lower doses associated with return to baseline after 1 week), effects on contraction frequency (doubles by week 3) and variability (decreases), electrophysiological disruption (decrease in beat duration, Ca^2+^ transients, depolarization duration), effects on ventricular function (fractional shortening decreased by ~30% with 100 ng/mL)	[98]
CS (IFN-γ, poly(I:C), IL-1β and endothelin-1)	Magnitude of inflammation dependent on organoid vascularization, hVCOs (CS) exhibit an increase in time to relaxation and preservation of contractile force, hVCOs (Endothelin-1) exhibit increased time to relaxation with increased contractile force and increased rate of contraction (diastolic contractile dysfunction), effects mitigated by endothelin-1 antagonists (bosentan, sitaxsentan)	[53]
